# Adaptations of the Walking Corsi Test (WalCT) for 2- and 3-year-old preterm and term-born toddlers: A preliminary study

**DOI:** 10.3389/fped.2023.1081042

**Published:** 2023-03-17

**Authors:** Nuria Martín-Pozuelo, Verónica Robles-García, Laura Piccardi, Alejandro Quintela del Rio, Javier Cudeiro, Isabel De las Cuevas-Terán

**Affiliations:** ^1^ Neuroscience and Motor Control Group, Department of Physical Therapy, Medicine and Biomedical Sciences, Biomedical Institute of A Coruña (INIBIC), Universidade da Coruña, A Coruña, Spain; ^2^ Escuelas Universitarias Gimbernat, University of Cantabria, Torrelavega, Spain; ^3^ Department of Psychology, Sapienza University of Rome, Rome, Italy; ^4^ San Raffaele Cassino Hospital, Cassino, Italy; ^5^ Department of Mathematics, Universidade da Coruña, A Coruña, Spain; ^6^ Neonatal Unit, Pediatric Department, Marqués de Valdecilla University Hospital, Research Institute Valdecilla (IDIVAL), Santander, Spain; ^7^ Department of Medical and Surgical Sciences, University of Cantabria, Santander, Spain

**Keywords:** spatial navigation, child development, selective attention, locomotion, premature infant, toodler

## Abstract

**Introduction:**

Topographical memory is crucial for navigation and environmental representation. The Walking Corsi Test (WalCT) has been used to evaluate topographical memory in children from 4 years upward. The present study aims to determine whether adapted versions of the WalCT- by simplifying instructions and increasing motivation- can be adopted to test topographical memory in 2- and 3-year-old toddlers born at term and preterm. Assessing this skill in such young children is important in light of recent studies that have shown how spatial cognition underlies the development of skills in other cognitive domains as well. Methods: For this purpose, 47 toddlers (27.39 ± 4.34 months, 38.3% females), 20 born at term and 27 preterm, performed two aimed-designed versions of WalCT.

**Results:**

The results showed better performance of the term groups with increasing age and for both versions. On the other hand, performance was better in 2-year-old term toddlers vs. preterm. When rising motivation, 2-year-old preterm toddlers improve their performance but differences between both groups were still significant. The preterm group showed lower performance related to lower levels of attention.

**Discussion:**

This study provides preliminary data on the suitability of the adapted versions of WalCT in early ages and prematurity conditions.

## 1. Introduction

Locomotion is the motor action that allows body displacement in space. In humans, it starts to develop within the first year of life during activities such as crawling or walking (1, 2). From the beginning of those, infants move purposefully. The development of motor control as well as proprioceptive, vestibular, visual, or cognitive functions (3, 4) allow them to learn to navigate in the environment (5–7). Functional locomotion is characterized not only by horizontal displacement but also by turns of the body. Therefore, elements such as head and gaze anticipation or axial postural synergies are essential, as they orient the body in space (by visual and vestibular systems) and determine trajectories using external spatial references (4, 8).

The maturation of elements of locomotion and navigational skills occur gradually. Visuomotor eye-hand integration generates the first relationship between the body and the external space (1, 9) and allows egocentric coding of external spatial references at a grasping distance (10, 11). By the age of 6–9 months, infants orient in the environment using only egocentric strategies (12–14). Then, references of an object in space start to be externally taken (15). Allocentric processing builds up from approximately 11 months when infants can use landmark information (14), and 18 months when they can reorient themselves (16–20). Both egocentric and allocentric frames must be integrated to elaborate internal spatial representations (21, 22). However, other spatial cognition abilities, such as spatial memory, planning, and learning, need to be developed and refined to navigate successfully (5). The whole development process starts early, but navigational skills are not consolidated until 9–10 years old. It is believed that they are not mastered until early adolescence (4, 8). Nevertheless, Boccia et al. (23) demonstrated that after a 12-week navigational training program, 4- to 5-year-old children significantly improved their performances achieving the acquisition of a map-like representation that is typically achieved by age seven. This finding suggests that navigational skills can be acquired earlier and that they likely underlie other skills.

Undoubtedly, topographic memory is crucial since it allows the coding and maintenance of online topographical information while updating the perspective of each new orientation, helping to generate useful trajectories to walk to reach a goal (5, 15, 24). Topographic memory is a component of visuospatial memory devoted to storing and recalling environmental information as well as body movements in the environment. The spatial relationships between landmarks also consider the individual's position in the environment *per se*. Pickering and colleagues reported dissociations between visual and spatial components that seemed to develop at different rates with little overlap in a given age group of children (25). These authors also suggest that a distinction should be made between the static and dynamic nature of visual and visuospatial stimuli to explain the steeper developmental increase in static than in dynamic performance due to the geometric knowledge acquired with schooling. Piccardi et al. (15) found that topographic memory also develops at different rates for stimuli located in the space of reaching vs. those present in the walking space. However, these authors investigated topographic memory through the Walking Corsi Test (WalCT) in children from 4 years of age without considering the youngest. The WalCT was developed to test topographic memory by Piccardi and colleagues (26, 27), who scaled to the extrapersonal navigational space the well-known and widely used in clinical and experimental settings Corsi Blocking Tapping Test (CBT) (28–30). The WalCT comprises nine black squared tiles placed on a carpet in a specific scaled layout (like CBT). By walking a predefined trajectory (squared tile sequence), the examiner demonstrates that the individual observes and then reproduces (15). Participants obtain a squared tile span corresponding to the longest sequence remembered correctly. WalCT has been extensively used in different populations with and without pathologies (27, 31–34) to assess memory of short paths in a vista navigational space, “the space that can be visually apprehended from a single location or with only little exploratory movements” (35).

Authors Piccardi et al. (15, 28) collected normative data both in adults and in children aged 4–11 years with the aim to provide a standard administration procedure to be used in clinical and educational practice (15, 36). Nemmi et al. (37) investigated the brain regions involved in the performance of WalCT finding an activation of the right dorso-lateral prefrontal cortex, calcarine sulcus and lingual gyrus, in particular the region in which the calcarine sulcus joins a part of the retrosplenial complex, a crucial region for solving navigational tasks (38–40).

The WalCT has shown a gradual and constant improvement in performance over age in typically developed children (15). Additionally, WalCT has been shown to be sensitive in detecting differences in performance compared with populations of children with cerebral palsy (5). Bartonek et al. (23), in particular regarding functional mobility, found that children at levels II-IV of the Gross Motor Function Classification System (GMFCS) obtained significantly smaller WalCT spans (i.e., the number of squares that the child could retain and recall in the short-term). These authors also found that participants able to walk outside in the community, regardless of the type of motor disability, have better topographical memory than individuals who do not walk outside and mobile through wheelchairs. These results suggest how important the integrity of motor development is in building the environmental knowledge underlying navigation, differently from adults in which motor impairment does not reduce topographical memory spans (34). Therefore, studying topographical memory shortly after learning to walk would make possible to understand better certain relationships between typical motor development and spatial orientation to intervene early, especially in the case of preterm children.

Although Bartonek and colleagues (5) did not determine what aspect could contribute more to spatial cognition deficits, they agreed that prematurity could influence, as stated also in other studies (41–43). Fernandez-Baizan et al. evidenced how 22 months old preterm infants performed worse than their peers when remembering a location and updating learned information (43). Also, 5–6 years old children born preterm with low birth weight have difficulty orienting themselves using an allocentric strategy (42). Besides, the literature describes poorer selective attention and worse visuospatial, visuomotor and fine motor performance in preterm-born infants without major neurological dysfunctions (44–47), which might affect topographic working memory. Additionally, it has been reported that when those alterations persist over time, they could also have an impact on school performance and integration with peers (48).

In addition, in 2010 Iaria et al. (49) described a neurodevelopmental disorder that impairs spatial cognition, the developmental topographic disorientation (DTD), that undermines the ability to orient in space in the absence of neurological damage or psychiatric disorders. Subsequent studies have shown that the disorder is widespread and present worldwide, making it even more important to assess navigational skills early in order to intervene to prevent the onset of navigational disorders (49–55).

In light of this evidence, this study aims to adapt the WalCT to measure topographic memory in preterm and term-born children under 4 years of age. Adapting a standardized instrument to early assess the different aspects of navigation is important to gain insights into specific features characterizing locomotion in preterm children and develop suitable early intervention programs if needed.

## 2. Methods

### 2.1. Participants

The present study was carried out in 21 born at term (T) Spanish toddlers (9 females and 12 males) and 28 preterm (PT) Spanish toddlers (10 females, 18 males). Two toddlers felt off the study because they were crying from the beginning of the task [*n* = 20 (T), *n* = 27 (PT)] (Table 1). Chronological ages ranged from 23 months to 39 months (mean age = 27.394 ± 4.34 months). Participants were subdivided into 3 groups according to age and prematurity: 12 born at term toddlers aged 23 to 24 months (5 females and 7 males), 8 born at term toddlers aged 38–41 months (4 females and 4 males), and 27 healthy preterm toddlers (70% with low birth weight) of 24 months old of corrected age (9 females and 18 males) who were born at 28–35 weeks of gestational age.

**Table 1:** Characteristics of participants.

	PT 2 years	T 2 years	T 3 years
Sample size (N)	27	12	8
Gender (Female%)	33.3	41.66	50
Race (% Caucasian)	76.92	91.67	100
Age (Mean, SD)	24 (0) *corrected*	23.58 (0.51)	36.37 (1.06)
HL language alarm signs (none %)	100	n.a.	n.a.
FMQ-PDMS-2 (Mean, SD)	86 (9.90)	86.13 (7.51)	n.a.
Mean TBCT omissions (SD)	10.69 (4.44)	3.41 (2.9)	0.62 (0.74)

PT, Preterm; T, Term; HL, Haizea – Llevant Table; FMQ-PDMS-2, The Fine Motor Quotient of the Peabody Developmental Motor Scale, second edition. TBCT, Teddy Bear Cancellation Test; n.a., not applicable.

Preterm group was followed-up from birth until 24 month-old by a neonatologist, co-author of the study. Haizea-Llevant, a standardized screening tool for social, cognitive and motor development in children from 0 to 5 years old, was used (56). Medical assessment during hospital stay in the neonatal period also comprises a neurological exam (cranial ultrasound, retinal assessment, evoked potentials -for infants under 1,500 grams or with risk factors for deafness- and otocheck hearing screen). Co- morbidity variables were registered, only 3 of the PT total sample scored under 7 in Apgar-5, 3 suffered bronchopulmonary dysplasia and 2 needed mechanic ventilation during their stay in the neonatal unit. None of them had visual or hearing impairments or had been diagnosed with neurological conditions. The Fine Motor Quotient (FMQ) of the Peabody Developmental Motor Scale-2 (PDMS-2; 57): was used to ensure there were no alterations in visuomotor and grasping abilities for all the preterm toddlers. FMQ was also checked with FMQ-PDMS-2 in a convenient sample of 8 toddlers from the control group. Other aspects of developmental alarm signs (language and comprehension levels) were asked to caregivers and there was no report of any disturbances in any group (Table 1).

This study was conducted following the Declaration of Helsinki. Ethical approval was granted from the ethical committee of Ethical Clinical Research Commitee of Cantabria (IDIVAL) with reference 10/2017. Parents of all toddlers signed informed consent before the assessment.

Data were collected at two different places: at a nursery in A Coruña (*n* = 21 T) and at a hospital in Marqués de Valdecilla University Hospital (*n* = 28 PT). Both were familiar environments for the children, as they often frequented them. Even so, the layout of rooms was the same; the testing space was covered by curtains, eliminating distractors from walls and floor and set up in a quiet and empty room. Parents and/or teachers were present in the room without interacting during the testing. Two trained pediatric physical therapists performed all evaluations. Preterm children were also evaluated by a neonatologist in the follow-up program from discharge.

### 2.2. Instruments and procedure

Topographic memory was evaluated using two purpose-designed adaptations of the original WalCT (15, 26, 27): The Adapted Walking Corsi Test and the Treasure Adapted Walking Corsi Test. Additionally, visuospatial attention was evaluated with the Teddy Bear Cancellation Test (60) to assess its contribution to navigational tasks.

#### 2.2.1. Adapted Walking Corsi Test (AWalCT)

**Figure 1:**
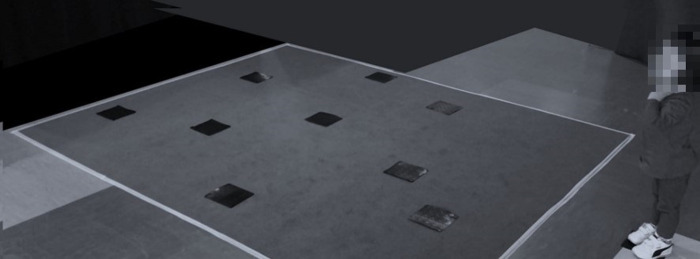
Child performing AWalCT. The toddler stands in front of the reduced carpet waiting The Examiner to start the task.

To adapt the WalCT for toddlers under 4 years old, we reduced the total size of the carpet by 61.33% (230 × 200 cm) and the size of the squares (from 30 × 30 to 10 × 10 cm) considering that children's navigation is strongly influenced by the characteristics of the environment (i.e., presence/absence of landmarks; size of the environment) (58): (Figure 1).

Additionally, instructions were simplified. From the starting point, the examiner showed a specific sequence by walking from one square to another. Toddlers, standing at the starting point, were asked to look attentively during the demonstration and imitate the examiner by walking and reaching the different squares arranged on the floor in the same order shown. Caregivers were placed close to them at the starting point and encourage them to participate. Examiners controlled visual engagement of the task demonstration and if this got lost, examiner repeated the demonstration and the instructions over again. Children were motivated every trial by examiners and caregivers (reward messages such as “well done” and applause).

Once the examiner went outside the carpet without stepping on any other tile so as not to confuse the child, the child was asked to do the same. This procedure was repeated up to three times to insure comprehension of the task.

The test started with only one square to reach; if the toddler did it correctly, the examiner increased the number of squares to reach [sequence length (SL)]. The first 3 random pretrials were used to familiarize toddlers with the task. Participants had to perform 3 out of 5 different sequences for each SL to pass to the next. Sequences administered were standardized and are reported in the Supplementary material. The task finished when the child was not able to complete 3 different sequences within an SL. Permutation errors, violations of serial order, omissions, or deviations from the correct path, were not allowed.

Participants obtained a square span (SS) corresponding to the longest sequence they could successfully perform. Additionally, we included the following related variables in the original WalCT:

- Starting (S), indicating whether the toddlers could initiate the task or not. Instructions were repeated up to three times, leaving 2 min in-between for the children to start the task.
- Composite score (CS), indicating the accuracy (fewest possible attempts) when performing the task. CS was calculated from the number of consecutive trials of the same SL. If the toddler correctly performed the first three consecutive sequences within an SL scored 5 points. If the toddler performed three correct sequences in 4 attempts, 4 points were scored. Finally, if the toddler completed three correct sequences in 5 attempts, it scored 3 points.
- Sum of the composite score (SCS) indicating the sum of composite scores at all SL reached. If the child obtained 5 points in SL 1 and 3 points in SL 2, the SCS was 8.

Regardless of the outcome, children were always verbally praised and after AWalCT a 5 minutes rest was provided to start the next adapted version (TWalCT).

#### 2.2.2. Treasure Walking Corsi Test (TWalCT)

The TWalCT was designed to increase toddlers' motivation during the task. The TWalCT was similar to AWalCT and had the same instructions but added rewards under the squares. The rewards were small toddlers’ toys. The examiner hid the rewards while walking from one square to another, controlling visual engagement. The demonstration of the task could be repeated up to three times. Then, toddlers were asked to walk and reach only at the locations where the rewards were hidden and in the same order. Rewards had different colors to make the task easier. Again, after 3 random pretrials, the test started with only one square to reach; and followed by increasing the number of squares (SL). Three out of 5 different sequences for each SL were needed. Sequences administered were different from the AWalCT but similarly standardized. If the child did not manage to find the rewards following the path marked by the examiner in a total of 3 different sequences within the same SL, the test was no longer valid. Anyway, at the end, examiners help the child to find all the rewards in a playful way, just to keep it as a positive experience. We considered SL, SS, S, CS, and SCS variables as described above.

#### 2.2.3. Teddy Bear Cancellation Test (TBCT)

The Teddy Bear Cancellation Test [TBCT (59)] is a useful test to study children's attention. Fifteen teddy bear pictures were presented on a A4 paper among 60 distractors (other pictures of toys, such as a ball, a car, a doll, etc.). The examiner sat in front of the toddler and asked the toddler to look carefully at the sheet and search the fifteen teddy bears by pointing at them. Finger painting was used to cross out the teddy bears and facilitate accounting of the teddy bears found. The child was told the following: “*On this sheet of paper there are a lot of drawings, some of them are teddy bears like this one* (pointing to a teddy bear). *Let*’*s tap all teddy bears you see, like that*” (showing a crossed out teddy bear as an example). Scores were calculated based on the number of omissions (NO), indicating the number of teddies not crossed out. The test was finished when all the teddy bears were cancelled or when the child stopped. The examiner asked once if the task was finished and waited the toddler to follow or said, *“There are no more teddy bears*”. This test has been used in children from 3 to 7 years old, however, simple instructions and tasks allowed to use it for younger children in this study.

### 2.3. Statistical analysis

Regarding the descriptive and inferential data analysis carried out, the Mann–Whitney U test for the assessment of significant differences in the quantitative variables (SS, CS, SCS, NO) was employed because the normality assumption could not be accepted for them (using the Shapiro–Wilk test). With the categorical variables (S), the binomial test was applied to the dataset to determine if the sample proportion could be considered adequate for the general population. Spearman's correlation was used to measure the correlation between numerical variables because of their discrete values. The significance level was set at *p* < 0.05.

Due to the small sample in most of the subgroups, significant and nonspurious differences were also analyzed by means of Bayesian hypothesis testing (60). A Bayesian test proceeds similarly to a classical or frequentist hypothesis test, but the *p*-value for making a decision is replaced by the named Bayes factor (BF). This number is the probability ratio of the null hypothesis and the alternative, conditioned by the statistical information provided by the sample data. If BF = 1, both the null and alternative hypotheses have the same level of credibility. If BF > 1, the data are more likely under the alternative hypothesis than under the null hypothesis. In this work, the obtained Bayes factors were interpreted using the classification by Taylor and colleagues (61), where the strength of the probability is graded as follows: 1–3, anecdotal evidence; 3–10, moderate evidence; 10–30, strong; 30–100, very strong; and > 100, extreme. Effect sizes (Cohen’s d) were computed directly from the data.

All classical statistical analyses were performed using SPSS software, and Bayesian tests were performed with R software.

## 3. Results

Binomial tests showed that not all toddlers could start AWalCT. There was a large difference between T and PT toddlers. While 2-year-old T toddlers tried to perform the task 83% of the time (*p* = 0.039 in the binomial test, Bayes factor BF = 3.8) (d = 0.33), only 22% of 2-year-old preterm infants performed it (*p* = 0.006, BF = 15.05) (d = 0.28). This means that the rest of the variables of AWalCT in the PT group cannot be statically analyzed because of the lack of data during the task (*n* = 6), since other PT children could not even start). However, this was different at TWalCT; all 2-year-old T (100%, *p* = 0.00) (d = 0.5) and the majority of PT toddlers could start the task (78%, *p* = 0.0059, BF = 15.05) (d = 0.27).

T toddlers who started AWalCT achieved span 1 at 24 months (*p* = 0.002, BF = 28.11) (d = 0.35), but only three out of 6 PT toddlers could achieve span 1. For preterm toddlers reaching span 1, the results on the CS and SCS were similar to the results in the T group.

TWalCT was designed to make AWalCT easier by increasing motivation. The results obtained were different between the two versions. Interestingly, 2-year-old preterm toddlers improved their performance during TWalCT because the number of toddlers who tried to perform the task was higher, as mentioned before, but also because some of them achieved span 1 (67%). However, there was still a significant difference between groups (*p* = 0.014, BF = 4.76) (d = 1.12), as 100% of the 2-year-old T group achieved a span of 1 or more during TWalCT. Regarding other variables, CS and SCS were significantly higher in the 2-year-old T group (*p* = 0.04, BF = 2.4, d = 1.06, and *p* = 0.01, BF = 6.21, d = 1.25, respectively), which means that all these variables are useful to detect differences between T and PT groups when performing the TWalCT.

3-year-old T toddlers improved both AWalCT and TWalCT execution. SS in AWalCT significantly improved from 1 to 2.14 (2 years - 3 years; *p* = 0.005 BF > 100) (d = 2.15) and in TWalCT from 1.08 to 2.00 (*p* = 0.010, *p* = 0.000 BF = 96) (d = 1.86). Additionally, SCS in AWalCT significantly improved from 4 to 9.43 (*p* = 0.10 *p* < 0.001 BF > 100) (d = 2.36). Most other variables also improved but not significantly. It seems that age influences performance to some extent. The comparison between AWalCT and TWalCT in T toddlers at different ages showed no significant differences in any variable.

Visuomotor and grasping abilities using the FMQ-PDMS-2 did not show any difference between groups (*p* = 0.974). Spearman analyses did not point to any correlation of FMQ-PDMS-2 with variables of TBCT, AWalCT or TWalCT.

To study the role of attention in topographical memory outcomes, we studied the TBCT. This analysis showed that PT toddlers pay less attention (have more number of omissions) during TBCT than matched-age T toddlers. Additionally, TBCT was better in 3-year-old T toddlers than in 2-year-old T toddlers (*p* < 0.001, BF > 100) (d = 0.33).

**Figure 2:**
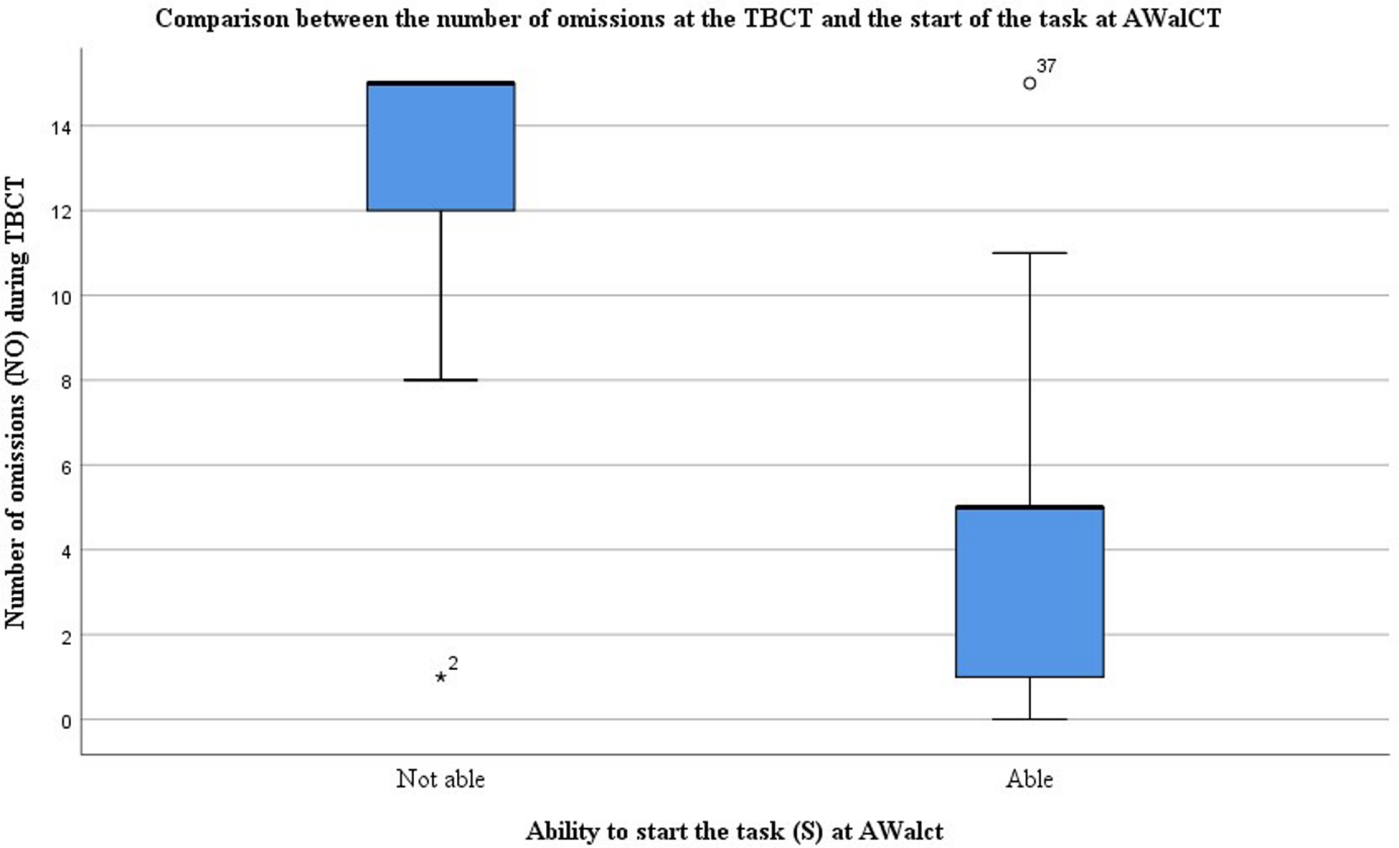
Boxplot comparing the number of omissions at the TBCT and the start of the task at AWalCT. The graph shows marked differences between subjects able and unable to start the task, being the median values of 5 and 15 omissions, respectively.

The Spearman correlation analyses showed an inverse moderate to strong correlation between the number of omissions at the TBCT and the start of the task at AWalCT (−0.714, d = 0.71). That is, the less attention the TBCT scored, the lower the probability of starting the AWalCT. In Figure 2, a boxplot of this variable for able and not able subjects to start AWalCT task illustrates the dissimilarity between the two groups and the negative correlation mentioned above. Correlations of AWalCT variables were not studied due to the lack of sample size.

On the other hand, correlations found at TWalCT were weak in S (−0.337, *p* = 0.020) (d = 0.33) and CS (−0.321, *p* = 0.043) (d = 0.32) and moderate in SS (−0.624, *p* = 0.000) (d = 0.62) and in SCS (−0.641, *p* = 0.000) (d = 0.64).

## 4. Discussion

The present study aimed to determine whether two newly adapted versions of WalCT (AWalCT and TWalCT) could be used to evaluate spatial navigation in 2- and 3-year-old children with easier instructions, reduced surface, and number of squares (AWalCT), and age-matched motivation elements (TWalCT). Additionally, we wanted to explore differences between born at term and preterm children since prematurity has been described as a risk factor for visuospatial alterations related to navigation.

Ability to start the task (S) at AWalCT is not useful to study topographic memory in 2-year-old preterm toddlers with low levels of attention. However, TWalCT can be used to evaluate topographic memory and detect differences between term and preterm groups. Additionally, the performance of children born at term depends on age. In both adapted versions of WalCT, added variables to the original WalCT gave valuable information to better understand topographic working memory.

### 4.1. Differences related to age in the two adapted versions of WalCT

The adapted versions of WalCT are useful for studying topographic memory in 2- and 3-year-old toddlers, confirming previous studies that describe how spatial navigation abilities emerge early in infancy (14). This study has similarities with other studies, such as Ribordy’s et al. (62), who evaluated allocentric spatial memory abilities in children from 18 months to 5 years by seeking and hiding a food reward in different locations in an open-field arena. These authors concluded that children after 25 months are capable of using allocentric spatial memory to identify and find a single location of a reward. In line with this study, 2-year-old children in the present research could achieve span 1, finding 1 location of the test when a reward toy was hidden. However, in our study, most 2-year-old typically developed children could also find a location without a reward. These data suggest that although topographic memory at 2 years old is still very simple, they can use it to explore and orient in the space, being better when strategies, such as hiding a reward, are used.

On the other hand, the performance of the two versions was better with age. Not only all children in the 3-year-old T group were able to initiate both tests but also because the execution was better, achieving greater sequence spans than the 2-year-old group. This finding is consistent with other studies using similar instruments, confirming the hypothesis that short-term spatial memory improves with age (62–64). In fact, in the original WalCT, Piccardi et al. tested 268 typically developing children from 4 to 11 years old and described a linear improvement in topographic memory over age (15). Therefore, it is consistent to think that this progression could behave the same in earlier stages. In this line, it would be interesting to know what happens before two years old; data indicate that children so young would not achieve span 0, at least in the absence of rewards.

Quantification of topographic memory in our sample was difficult since, in some cases, the span variable was not informative enough to discriminate differences between groups. Therefore, we added a composite score as a variable to study the number of errors that children make, as reported elsewhere (62, 63, 65). This type of measure gives us more precise information about the execution of the test. We found that the composite score was significantly better in the AWalCT in 3-year-old toddlers, meaning that they attained the targets in fewer trials than younger children. This is in line with other research affirming that there is an improvement in spatial discrimination ability in the period of two to three years (62).

Our data preliminarily demonstrate that AWalCT and TWalCT can be used to evaluate topographic memory in children 2 and 3 years old. The composite score is helpful to quantify the execution of those tests.

### 4.2. Toddlers born at term and preterm perform differently

Both versions of the WalCT showed different results between the preterm and born at term groups. Generally, children born at term executed better and could achieve span 1 or more, while preterm children could achieve span 1 at most. The underlying reasons for this cannot be extracted from our study. However, the relationship between preterm birth and visuospatial abilities has been described (41, 43, 45, 66–68) and could influence execution, taking into account that these abilities are essential to develop whole-body movements and move into the space (26, 27).

However, most of the literature referring to visuospatial deficits described them in children with cerebral lesions (41). No visual or neurological conditions were diagnosed in our sample, so this might not be the cause. Considering that the scientific literature argues that there are not enough tools to determine visual perception function at these ages objectively, it is likely that the mild visuospatial deficits in our sample may be unnoticed.

### 4.3. The role of attention in the study of spatial navigation abilities

This study adds relevant information regarding the influence of attention on assessing navigational tasks (69). Very importantly, attentional level measured by TBCT showed a moderate correlation with the sequence span obtained at the AWalCT and TWalCT. This type of relationship was already described in children but mainly focused on egocentric spatial skills such as reading (70–72). It is clear that visual selective attention needs to be present to some extent during WalCT performance, as children need to focus on the targeted squares by suppressing others that are not relevant (45). Therefore, the worse execution of the preterm group at both WalCT versions could be partially explained by the lower levels of attention. Previous studies have used cancellation tests similar to the D2 test, the NEPSY visual attention subtest or the Star Cancellation Task, detecting lower levels of selective attention in children born preterm (72–75). In a recent study by Faedda et al. (31), the importance of the attentional level in the execution of the standard WalCT in children affected by attention deficit hyperactivity disorder (ADHD) emerged. These children could recall a learned supra-span sequence after five minutes, but they showed difficulties in performing the topographic working memory task. Their deficits seem to be linked to difficulties in sustained attention, spatial memory for novel visual materials, poor visuospatial working memory, and perseverative behaviors.

However, as far as we know, selective attention was never measured with these tests below three years of age. Few studies describe that visual attention can primarily be evaluated before 2 years of life (76), and most studies start to evaluate it from 3 years of age, describing a better performance at approximately 6 years of age (59, 69, 73, 77).

We observed that preterm 2-year-old toddlers performed significantly worse than those born at term children in the present study. This latter had lower rates of omissions, decreasing with age, in agreement with the literature (44, 59, 78). Lower levels of attention affected topographic memory outcomes and children’s ability to start the task, as shown in the moderate to strong correlation between TBCT and this variable. Interestingly, this does not happen with the TWalCT, using object retrieval as a strategy to increase motivation (79, 80) and direct attention (21). Therefore, we estimate that TWalCT is more suitable for investigating the topographic memory of PT toddlers with low levels of attention.

### 4.4. Implications in early intervention

This is the first study to explore topographic memory in preterm-born toddlers before 3 years of age. However, their visuospatial difficulties have been widely studied and spatial memory has been described to be compromised (78). It is essential to determine whether the worse performance in attentional and navigational tasks of preterm-born toddlers shown here may be related to future problems such as low academic performance or participation, described elsewhere (81–83). If so, TWalCT could be suitable as a new tool to identify navigational disorders early, which has been strongly recommended (49). On the other hand, the possibility of using WalCT at such initial stages could be very useful for other conditions, such as cerebral palsy, of which early detection is known to be crucial to get started intervention as soon as possible and so, optimize outcomes (5, 84). Finally, since the ability to navigate improves with practice (41, 85), training through active spatial exploration in familiar and unfamiliar environments has been suggested as a targeted early intervention. This may positively impact preschool age (85, 86), preventing learning and behavioral problems.

### 4.5. Study limitations

Although our results are promising and present a new tool for assessing topographic memory in very young children, they must be interpreted with caution. First, this study was exploratory in nature, so more research is needed to validate AWalCT and TWalCT, analyze their psychometric properties with a power and sample size estimation and consider demographic factors that may affect navigation. Second, the lack of standardized tools for so young toddlers makes difficult to assess their navigational skills and other related such as selective attention. The TBCT has not previously shown data for 2–3-year-old children, so more research is needed in this aspect.

Finally, it would be necessary to know to what extent the low performance of the preterm group could be related to attention capacity or topographic memory itself and related to other alterations, such as developmental delay or visuomotor coordination impairments, at different stages. Also, comparisons related to gender or low birth weight have not been considered. However, the small sample size prevents such comparisons from being made, which should certainly be carried out on a larger sample, allowing for proper interpretation. Definitively, having more data could help us to size up the possibilities and need for early intervention.

## 5. Conclusion

The two versions of the original WalCT are suitable for evaluating topographic memory in 2- and 3-year-old toddlers with good attention levels. However, in children with lower levels of attention, AWalCT does not allow us to evaluate this navigational skill. Attention relates to navigational performance, as both improve with age and are poorer in the preterm population of this study. On the other hand, TWalCT, by increasing motivation, can be used in 2- and 3-year-old toddlers to detect differences between term and preterm children.

## Data Availability

The raw data supporting the conclusions of this article will be made available by the authors, without undue reservation.
