# Supplementary material for: Adaptations of the Walking Corsi Test (WalCT) for 2- and 3-year-old preterm and term-born toddlers: A preliminary study
Source: Front Pediatr. 2023 Mar 17;11:1081042. doi: 10.3389/fped.2023.1081042 (PMC10064058; doi:10.3389/fped.2023.1081042)

Walking Corsi Test for toddler (sequences by Piccardi et al 2013; 2014)

The examiner shows the sequence hiding a toy under each square soon after he/she stays on the square. Examiner stops on the square for 2 seconds.
After the examiner asks participant to repeat the same sequence previously showed walking exactly on the same squares. The blank map (below) needs to sign the path produced by the participant in such way it is possible to analyze the type of error. The examiner continues until the participant makes 3 out of 5 correct sequences of the same length. If participant is unable to make 3 out of 5, the examiner interrupts the task.

CORSI 1-A-

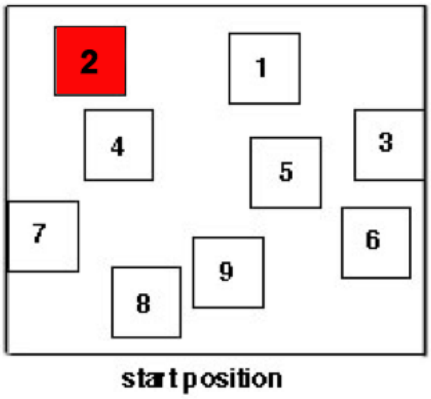

2

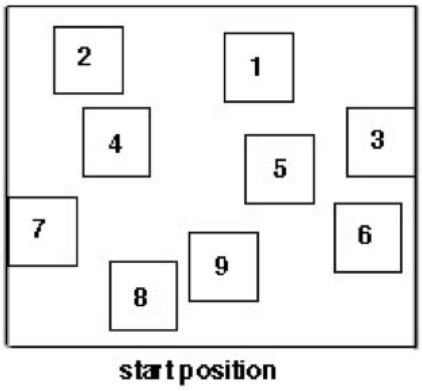

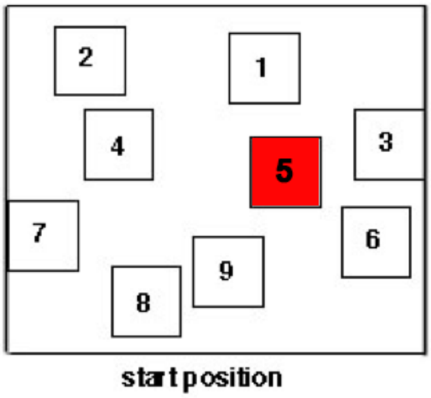

5

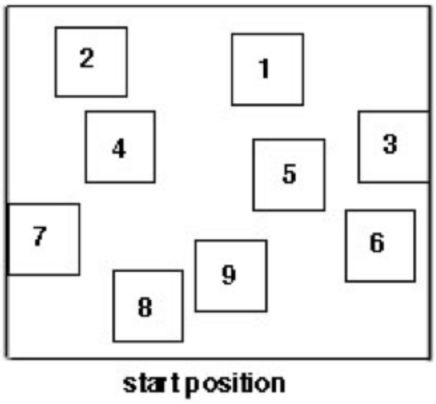

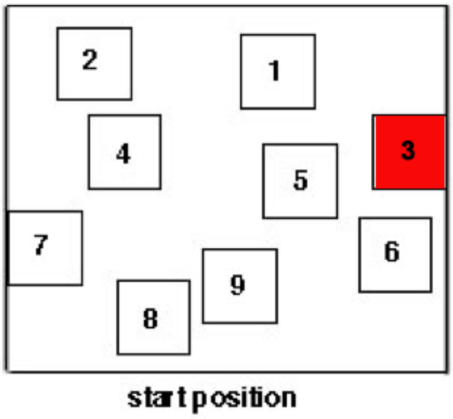

3

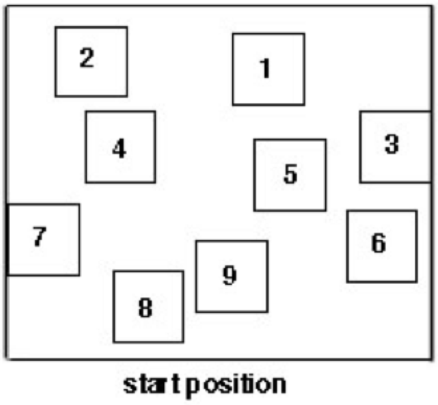

The square in red indicates the first tile to reach

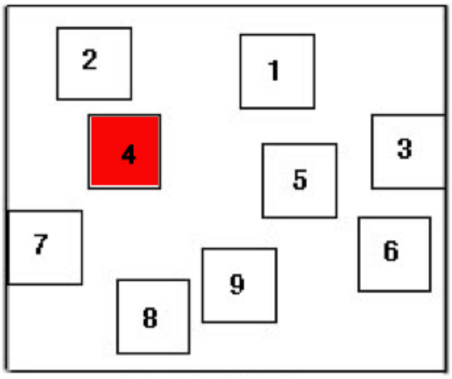

start position

4

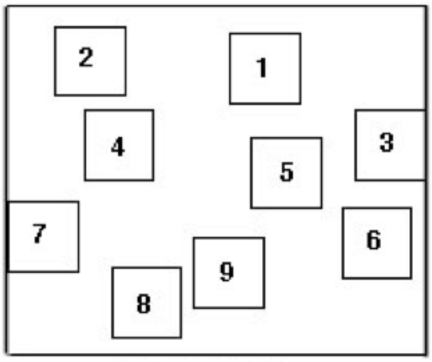

start position

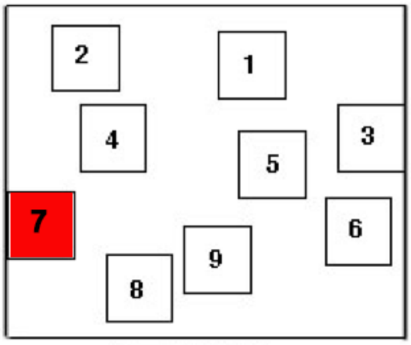

start position

7

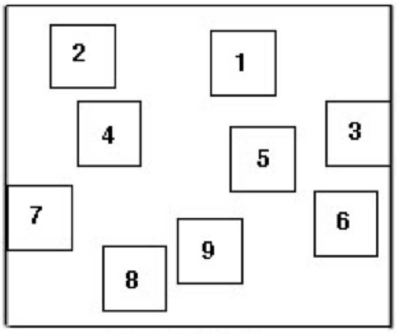

start position

CORSI 2 -A-

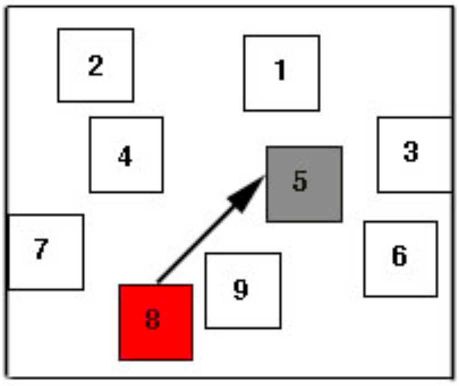

start position
(examiner + subject)

8-5

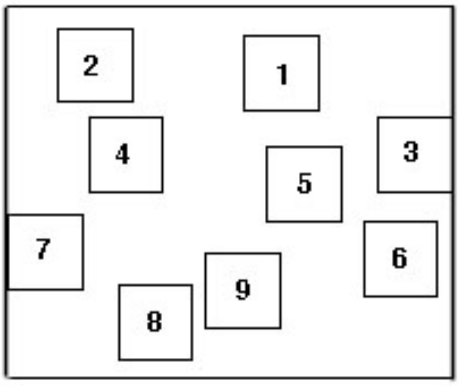

start position

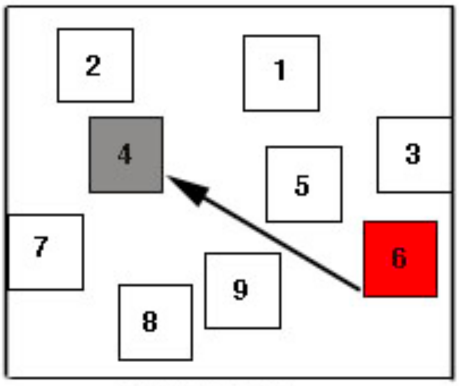

start position

6-4

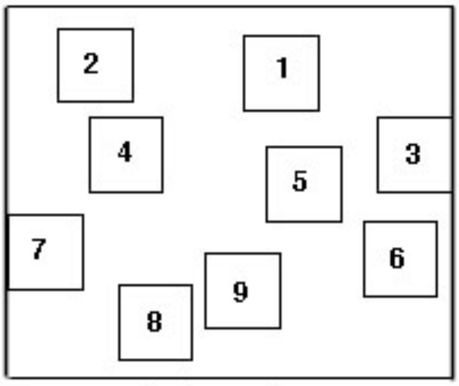

start position

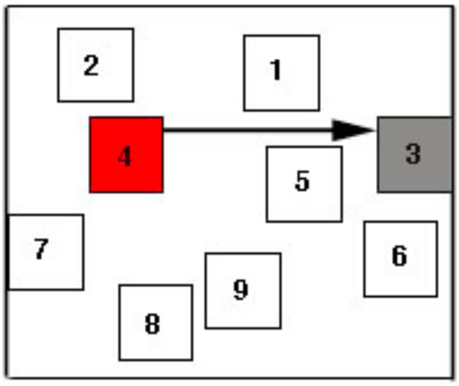

start position

4-3

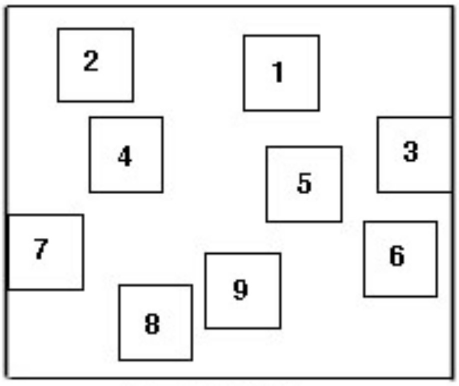

start position

CORSI 2 -A-
(continue)

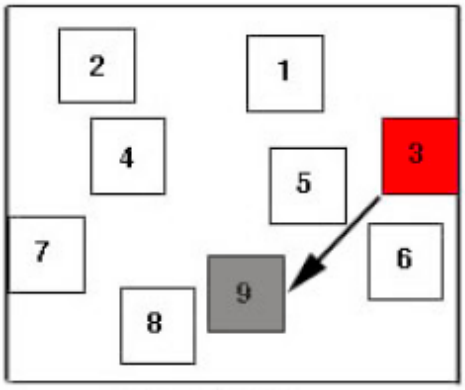

start position
(examiner + subject)

3-9

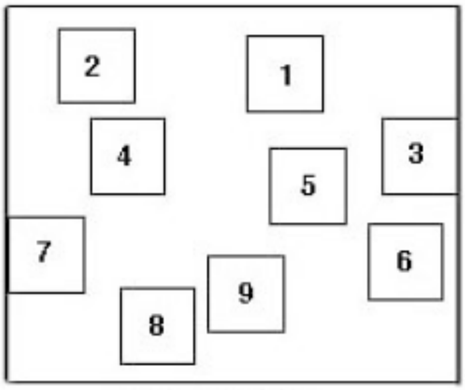

start position

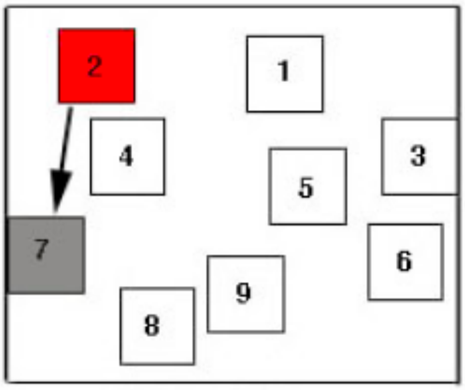

start position

2-7

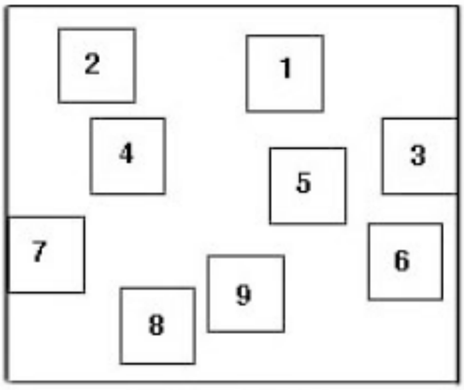

start position

CORSI 3 -A-

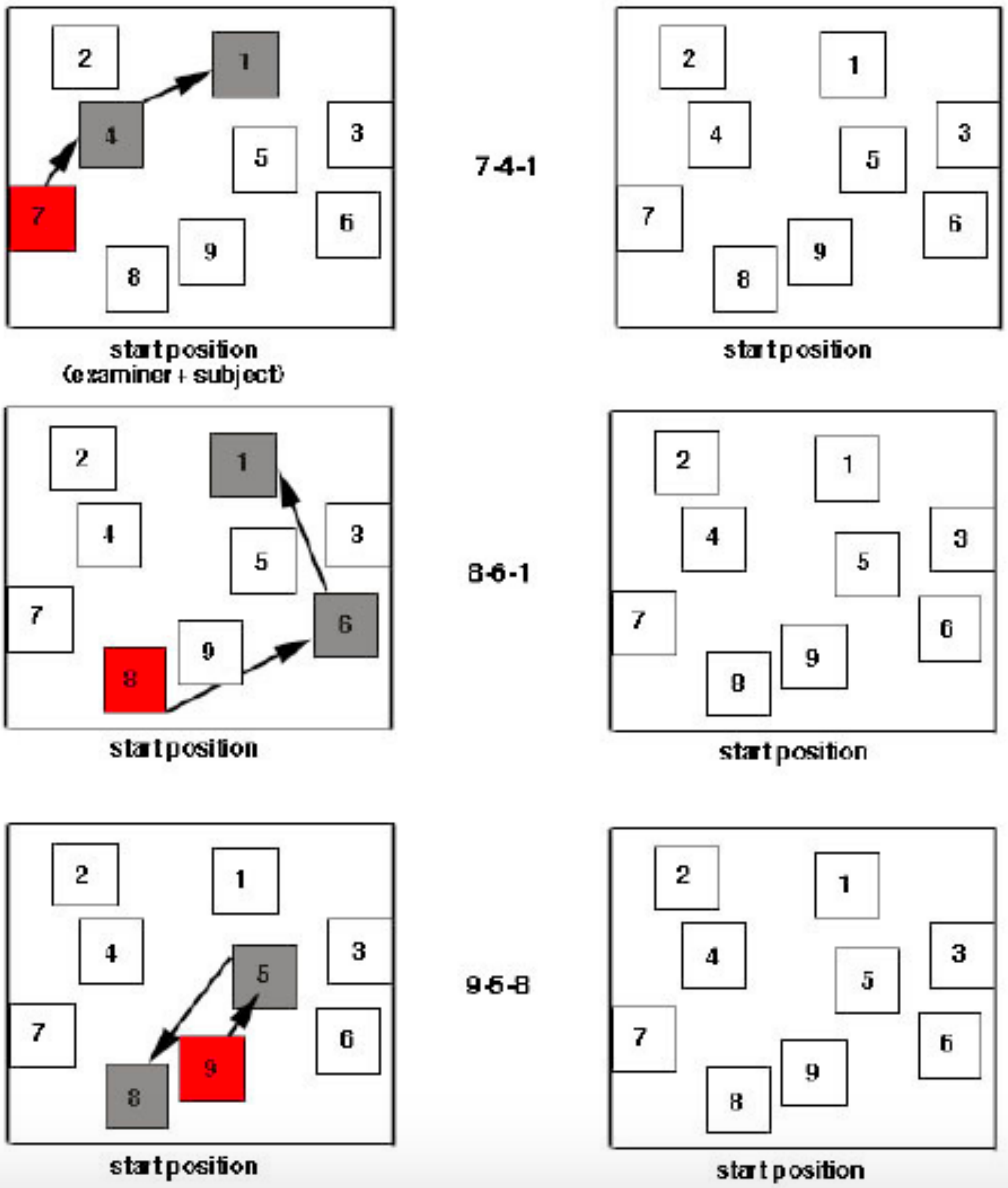
start position
(examiner + subject)
7-4-1
start position
start position
8-6-1
start position
start position
9-5-8
start position

CORSI 3 -A-
(continue)

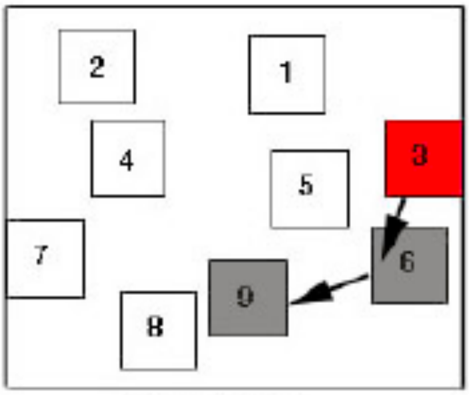

start position
(examiner+ subject)

3-6-9

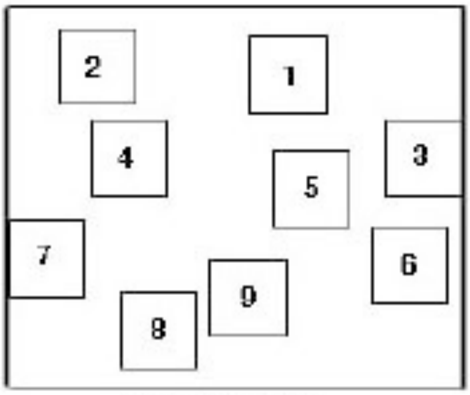

start position

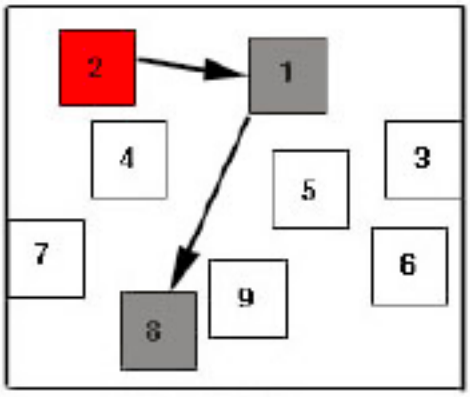

start position

2-1-8

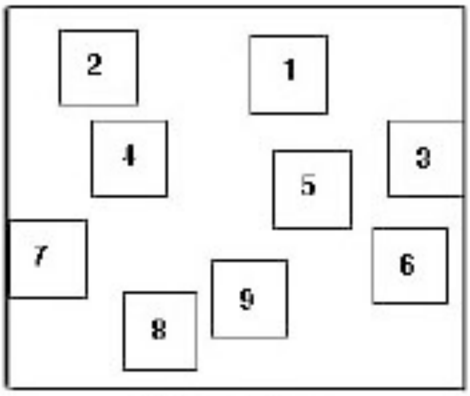

start position

CORSI 4 -A-

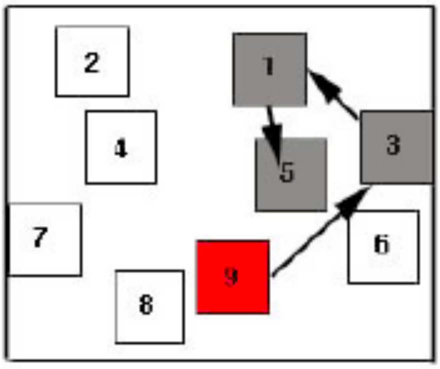

start position
(examiner + subject)

9-3-1-5

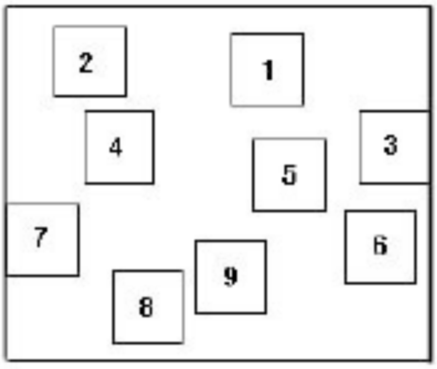

start position

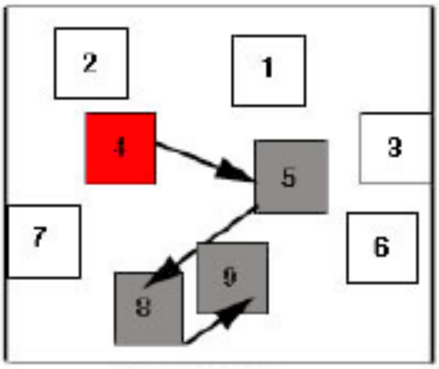

start position

4-5-8-9

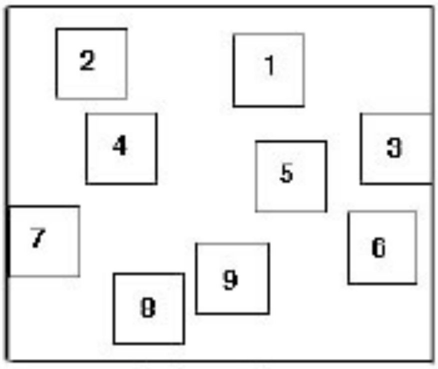

start position

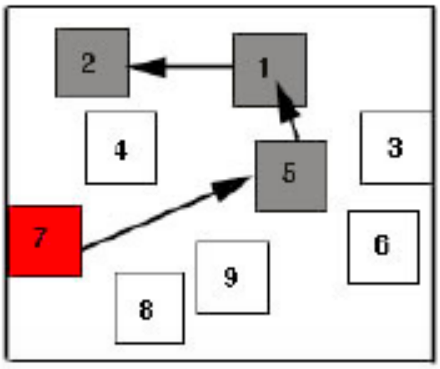

start position

7-5-1-2

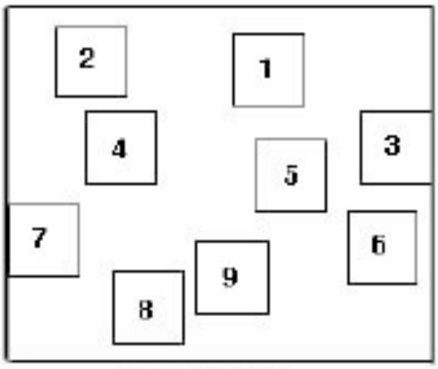

start position

CORSI 4 -A-

(continue)

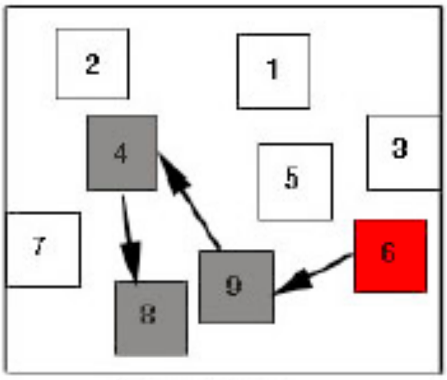

start position
(examiner+ subject)

6-9-4-8

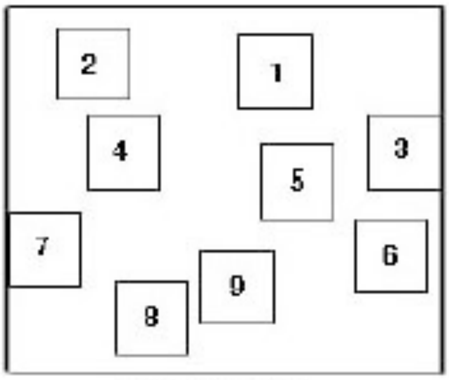

start position

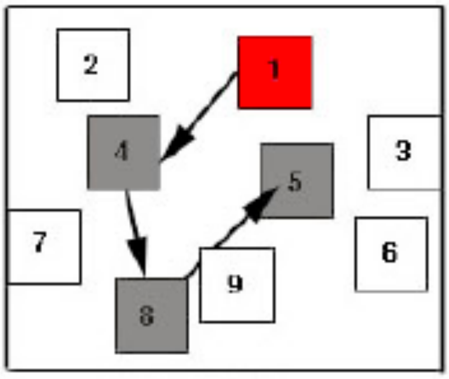

start position

1-4-8-5

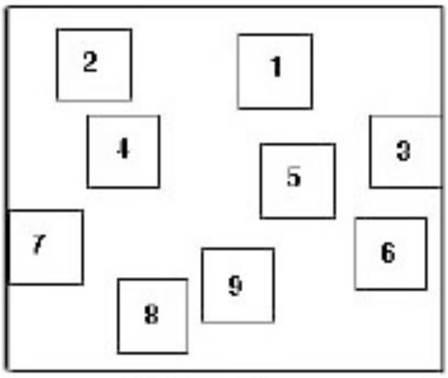

start position

CORSI 5 -A-

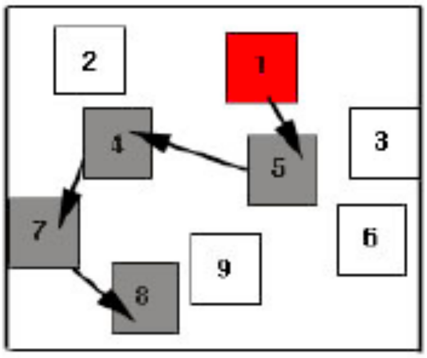

start position
(examiner + subject)

1-5-4-7-8

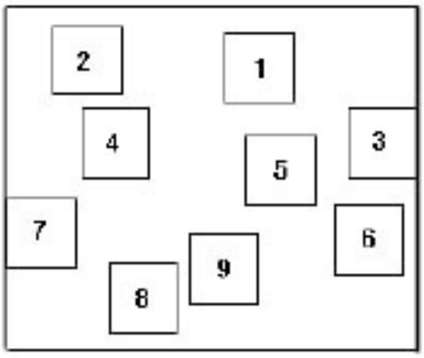

start position

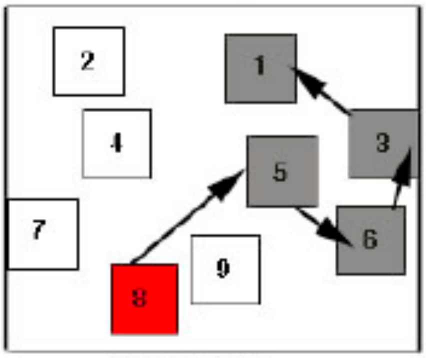

start position

8-5-6-3-1

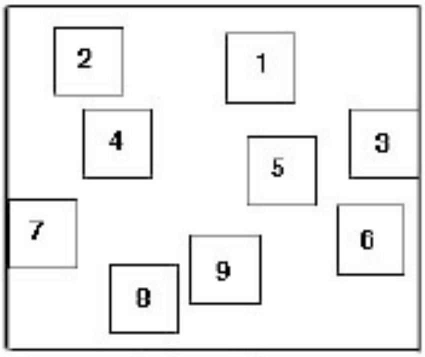

start position

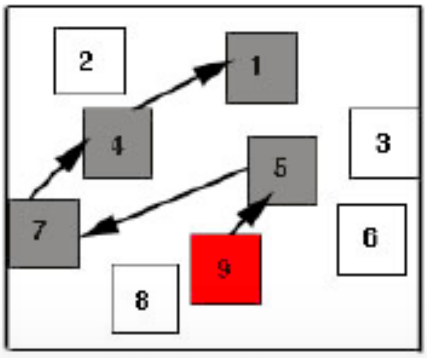

start position

9-5-7-4-1

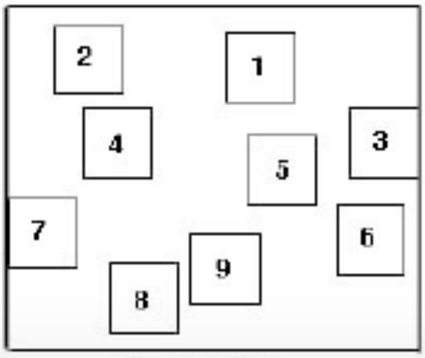

start position

CORSI 5 -A-

(continue)

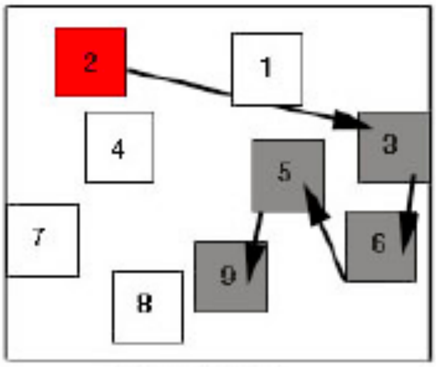

start position
(examiner + subject)

2-3-6-5-9

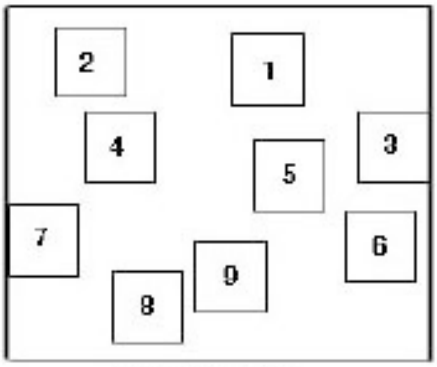

start position

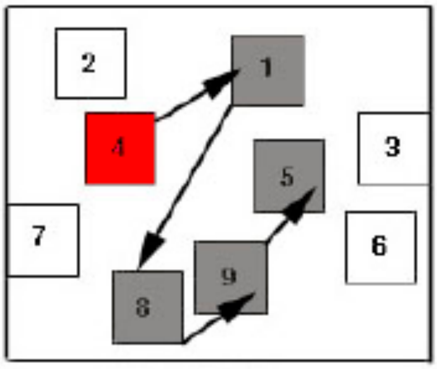

start position

4-1-8-9-5

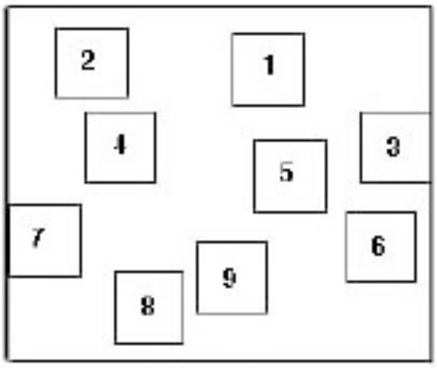

start position

GRASPING

Material: 2 bolitas de comida, cubos, sonajero, 2 folios

SUBTEST “GRASPING” PDMS2

**Item 1-Grasping Reflex**<br>**Edad: 0 meses**<br>Posición: Supino<br>Estimulo: Dedo del examinador<br>Procedimiento: Coloque al niño en decúbito supino. Estimule la palma de la mano del niño colocando su dedo índice en la palma de éste, junto al dedo pulgar.<br><br>Criterio:<br>2 Cierra la mano alrededor del dedo del examinador y aprieta.<br>1 Dobla los dedos ligeramente alrededor del dedo del examinador (hay un espacio entre el dedo del examinador y los del niño).<br>0 Extiende los dedos, no consigue doblarlos.	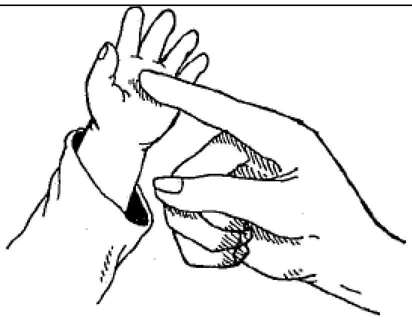
**Item 2- Grasping Cloth**<br>**Edad: 0 meses**<br>Posición: Supino<br>Estimulo: Toalla<br>Procedimiento: Extienda una **toallita** sobre su antebrazo y mantenga el brazo sobre el cuerpo del niño y a su alcance. Coloque la mano del niño en la parte superior de la toallita y observe su reflejo de prensión<br><br>Criterio:<br>2 Abre y cierra los dedos hasta que agarra la toallita.<br>1 Araña la toallita pero no la agarra.<br>0 Estira los dedos, pero no consigue agarrarla.	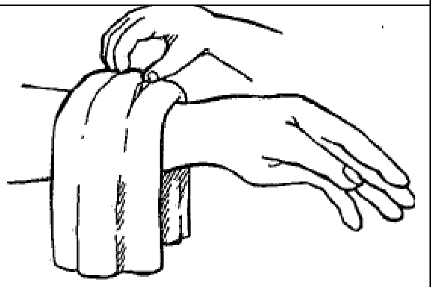
**Item 3- Releasing rattle-disappearing reflex**<br>**Edad: 0 meses**<br>Posición: Supino<br>Estimulo: Sonajero<br>Procedimiento: Coloque al niño en decúbito supino y ponga un **sonajero** en su mano. Después de que el niño haya sujetado el sonajero durante 5 segundos observe el tiempo que necesita para soltarlo.<br><br>Criterio:<br>2 Sujeta el sonajero durante 5 seg., y lo suelta en 3 seg.<br>1 Sujeta el sonajero durante 5 seg. y lo suelta en un plazo de 4 a 5 seg.<br>0 Sujeta el sonajero durante 5 seg. y lo suelta en más de 5 seg..	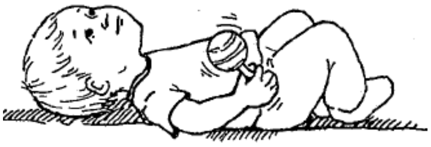

Item 4 - Grasping Rattle

Edad: 2 meses

Posición: Supino
Estimulo: Sonajero
Procedimiento: Coloque al niño en decúbito supino. Roce suavemente la palma de la mano del niño con un sonajero y diga : “coge el sonajero”

Criterio:
2-El niño agarra el sonajero
1-Toca el sonajero con los dedos pero no lo agarra
0-No consigue estirar los dedos

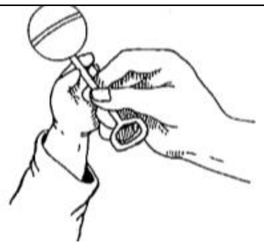

Item 5 – Holding Rattle

Edad: 2 meses

Posición: Supino
Estimulo: Sonajero
Procedimiento: Coloque al niño en decúbito supino. Ponga el sonajero en su mano.

Criterio: 2 El niño sujeta el sonajero durante 30 seg
1 El niño sujeta el sonajero de 15 a 29 seg
0 El niño sujeta el sonajero durante menos de 15 seg

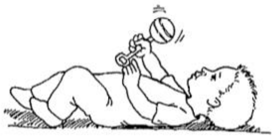

Item 6 – Manipulating Rattle

Edad: 3 meses

Posición: Supino
Estimulo: Sonajero
Procedimiento: Ponga al niño tumbado. Mueva el sonajero y luego póngalo en la mano del niño y diga “Mueve el Sonajero”

Criterio:
2-El niño mueve el sonajero 15 grados
1-El niño mueve el sonajero de 5 a 14 grados
0-El niño mueve el sonajero 4 grados o menos

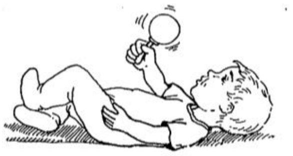

Item 7- Grasping Rattle

Edad: 4 meses

Posición: Sentado
Estimulo: Sonajero
Procedimiento: Siéntese en una mesa con el niño sentado en su regazo mirando hacia la mesa. Ponga un sonajero en la mesa a unos 7 cm de la mano del niño y diga “Coge el sonajero”

Criterio:
2-El niño coge el sonajero
1-El niño toca el sonajero
0-El niño extiende el brazo hacia el sonajero

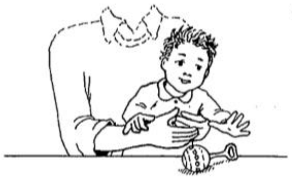

**<u>Item 8- Pulling String</u>**<br>**Edad: 5 meses**<br>Posición: Decúbito Prono<br>Estimulo: Juguete con una cuerda<br>Procedimiento: Ponga al niño decúbito prono. Ponga un juguete atado a una cuerda, en la línea media entre las manos del niño. Diga “Coge el juguete”<br><br>Criterio:<br>2-El niño coge la cuerda, tira de ella y obtiene el juguete<br>1-El niño agarra la cuerda, la toca o tira de ella<br>0-Mira el juguete	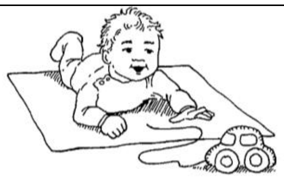
**<u>Item 9- Securing Paper</u>**<br>**Edad: 5 meses**<br>Posición: Sentado<br>Estimulo: Folio tamaño A4<br>Procedimiento: Siéntese en la mesa con el niño sobre su regazo, mirando la mesa. Ponga un papel a 7 cm de la mano del niño y diga “Coge el papel”<br><br>Criterio:<br>2-El niño obtiene el papel tirando de el con la palma de la mano o arrugándolo<br>1-Toca el papel<br>0-Estira la mano hacia el papel	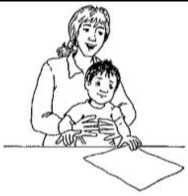
**<u>Item 10- Grasping Cube</u>**<br>**Edad: 5 meses**<br>Posición: Sentado<br>Estimulo: Un cubo<br>Procedimiento: Siéntese en la mesa con el niño sentado en su regazo mirando la mesa. Ponga un cubo en la mesa a 7 cm de la mano del niñoy diga “coge el cubo”<br><br>Criterio:<br>2-Agarra el cubo durante 15 seg<br>1-toca el cubo durante 15 seg<br>0-Estira la mano pero no consigue tocar el cubo	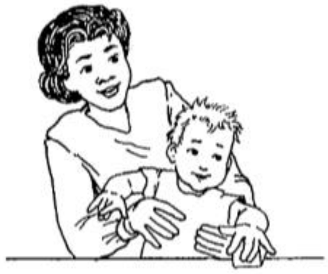
**<u>Item 11- Grasping Cube</u>**<br>**Edad:6 meses**<br>Posición: Sentado<br>Estimulo: Cubo<br>Procedimiento: Sientese en la mesa con el niño sentado en su regazo mirando la mesa. Atraiga la atención del niño al cubo que estará a 7 cm e su mano y diga “coge el cubo”. Observe como coge el niño el cubo<br><br>Criterio:<br>2-Agarra el cubo con los dedos anular y meñique y la palma de la mano, o con los dedos pulgar, índice y corazón<br>1-Agarra el cubo con el dedo meñique y la palma de la mano<br>0-Agarra el cubo con todo el puño	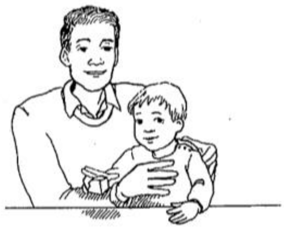

Item 12- Shaking Rattle

Edad:6 meses

Posicion: Sentado
Estimulo: Sonajero
Procedimiento: Sientese en la mesa con el niño en el regazo. Ponga el sonajero en la mano del niño y diga "Mueve el sonajero"

Criterio:
2-El niño sostiene el sonajero y lo mueve durante 60 seg
1-Mueve el sonajero de 11 a 59 seg
0-Mueve el sonajero durante 10 o menos seg

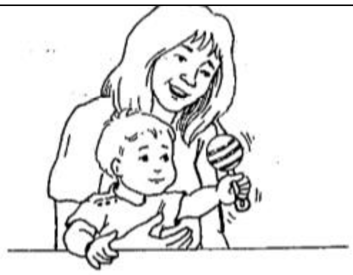

Item13- Shaking Rattle

Edad: 7 meses

Posicion: Sentado
Estimulo: Sonajero
Procedimiento: Sientese a la mesa y coloque al niño mirando hacia la misma y sentado sobre su regazo. Mueva el sonajero hacia atrás y hacia delante 3 veces describiendo un arco de 90 grados. Ponga el sonajero encima de la mesa delante del niño y diga "mueve el sonajero"

Criterio:
2-Mueve el sonajero en un arco de 90 grados 3 veces
1-Mueve el sonajero 3 veces en un arco de 45 a 89 grados
0-Mueve el sonajero menos de 45 grados o menos de 3 veces

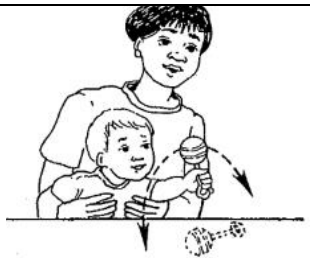

Item 14- Grasping Cube

Edad: 7 meses

Posición: Sentado
Estimulo: Cubo
Procedimiento: Siéntese a la mesa y coloque al niño mirando hacia la misma y sentado sobre su regazo. Ponga un cubo encima de la mesa a unos 7 cm de la mano del niño y diga: coge el cubo. Observe como coge el cubo el niño

Criterio:
2-Agarra el cubo con los dedos pulgar, índice y medio dejando un espacio
1-Agarra el cubo con los dedos índice y medio y la base de la mano (sin dejar espacio visible entre el cubo y la palma)
0-Agarra el cubo con todo el puño

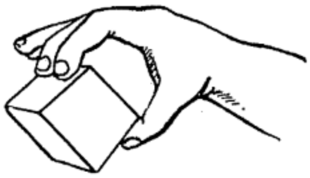

Item 15- Grasping Pellets

Edad:8 meses

Posición: Sentado
Estimulo:2 bolitas de comida
Procedimiento: Siéntese a la mesa y coloque al niño mirando hacia la misma y sentado sobre su regazo. Coloque 2 bolitas de comida juntas encima de la mesa al alance del niño. Diga "Coge toda la comida"

Criterio:
2-Agarra ambas bolitas a la vez arrastrándolas con los dedos (usando movimiento de dedos)

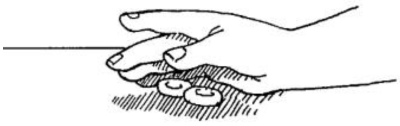

1-Agarra una bolita arrastrándola con los dedos
0-Toca una o ambas bolitas

Item 16- Manipulating Paper

Edad:8 meses

Posición: Sentado
Estimulo: Folio A4
Procedimiento: Siéntese a la mesa y coloque al niño mirando hacia la misma y sentado sobre su regazo. Ponga la mitad del folio en la mesa y diga: “Mira como arrugo el papel”. Arrugue el papel en una mano y coloque la otra mitad a unos 7 cm de la mano del niño. Dígale “Arruga el papel como lo he hecho yo”

Criterio:
2-Arruga el papel utilizando la palma de una mano o de las dos
1-Arruga el papel con los dedos
0-Toca el papel o tira de él

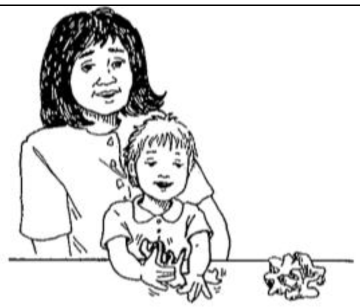

Item 17-Grasping Pellets

Edad: 8 meses

Posición: Sentado
Estimulo: 2 bolitas de comida
Procedimiento: Siéntese a la mesa y coloque al niño mirando hacia la misma y sentado sobre su regazo. Ponga 2 bolitas de comida juntas en la mesa al alcance de la mano del niño y diga: “Coge toda la comida”

Criterio:
2-Agarra las 2 bolitas arrastrándolas, con el dedo pulgar hacia el lateral del dedo índice semi flexionado, o agarra 1 bolita con el dedo pulgar estirado frente al lado interno del dedo índice (pinza inferior)
1-Agarra una bolita con los dedos pulgar e índice.
0-Agarra ambas bolitas arrastrándolas(movimiento de rastrillado)

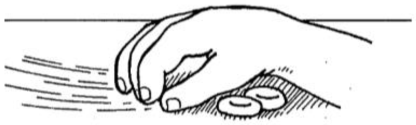

Item 18- Grasping pellets

Edad: 11 meses

Posición: Sentado
Estímulo: 2 bolitas de comida
Procedimiento: Siéntese a la mesa y coloque al niño mirando hacia la misma y sentado sobre su regazo. Ponga **2 bolitas de comida** en la mesa al alcance del niño. Diga: “coge toda la comida”.

Criterio
2 -Agarra 1 o 2 bolitas con las yemas de los dedos pulgar e índice, mientras mantiene la mano, la muñeca y el brazo fuera de la mesa.
1 -Agarra 1 o 2 bolitas con las yemas de los dedos pulgar e índice y mantiene el brazo apoyado en la mesa.
0 -Agarra una bolita usando un agarre que no sea con el dedo pulgar o la yema del dedo índice.

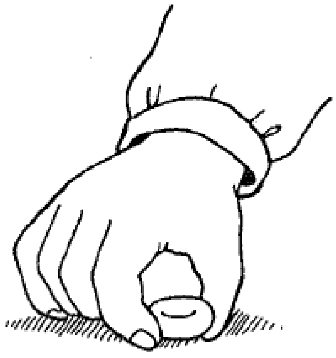

Item 19- Grasping cube

Edad: 11 meses

Posición: Sentado
Estímulo: Cubo
Procedimiento: Siéntese a la mesa y coloque al niño mirando hacia la misma y sentado sobre su regazo. Ponga un **cubo** a unos 7 cm de la mano del niño. Diga: "coge el cubo". Observe cómo lo coge.

Criterio
2 Agarra el cubo por la parte superior con el dedo pulgar en oposición a las yemas de los dedos índice y medio dejando un espacio visible entre el cubo y la palma de la mano.
1 Agarra el cubo por un lateral con el pulgar y las yemas de los dedos índice y medio (sin que la mano entre en contacto con la mesa).
0 Agarra el cubo con todo el puño.

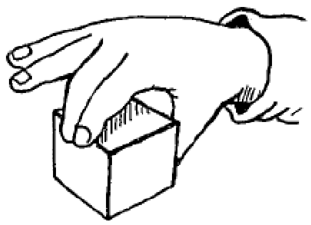

Item 20-Grasping cubes

Edad: 13 meses

Posición: Sedestación
Estímulo: 2 cubos
Procedimiento: Siéntese a la mesa y coloque al niño mirando hacia la misma y sentado sobre su regazo. Ponga **2 cubos** juntos y muéstrele cómo coger los dos cubos con una mano. Deje los cubos en la mesa y diga: "coge los dos cubos con una mano como hice yo".

Criterio
2- Agarra ambos cubos con una mano y los sujeta durante 3 seg.
1 -Agarra ambos cubos con una mano y los sujeta durante menos de 3 seg.
0 -Agarra un cubo.

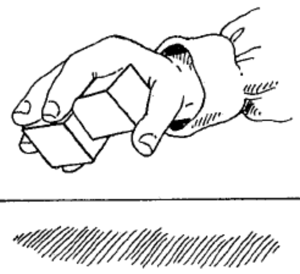

Item 21- Grasping Marker

Edad: 15-16 meses

Posición: Sedestación
Estímulo: Un rotulador y un folio DIN A4
Procedimiento: Ponga el rotulador y el folio en la mesa junto a la mano del niño. Diga: "dibuja una raya en el papel". Observe cómo agarra el rotulador.

Criterio
2 -Agarra el rotulador con los dedos pulgar e índice apuntando hacia el papel, y con los otros 3 dedos alrededor del rotulador.
1 -Agarra el rotulador con el dedo pulgar hacia arriba y con el dedo meñique apuntando hacia el papel.
0 -No consigue agarrar el rotulador.

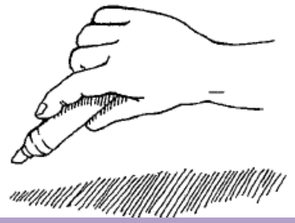

Item 22- Grasping Marker<br>**Edad: 41-42 meses**<br>Posición: Sedestación<br>Estimulo: Boligrafo y papel<br>Procedimiento: Ponga el **rotulador** y el **folio** en la mesa junto a la mano del niño. Diga: "dibuja una raya en el papel". Observe cómo agarra el rotulador.<br><br>Criterio<br>2 Agarra el rotulador con el dedo pulgar y la yema del dedo índice, dejando los otros 3 dedos apoyados sobre la palma de la mano. La parte superior del rotulador está apoyada entre los dedos pulgar e índice. Mueve la mano en bloque al dibujar.<br>1 Agarra el rotulador con el dedo pulgar y la yema del dedo índice; la parte superior del rotulador está apoyada entre los dedos pulgar e índice.<br>0 Agarra el rotulador con los dedos pulgar e índice.	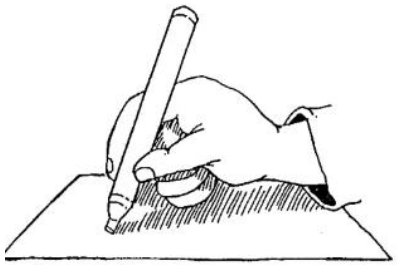
Item 23- Unbuttoning button<br>**Edad: 41-42 meses**<br>Posición: Sedestación<br>Estimulo: Banda de botones y cronometro<br>Procedimiento: Ponga la tira de botones en la mesa delante del niño. Diga "desabrochalos tan rápido como puedas"<br>Criterio<br>2 Desabrocha 3 botones en 75 o menos segundos<br>1 Desabrocha 3 botones en 76 o mas segundos<br>0 Intenta desabrochar los botones	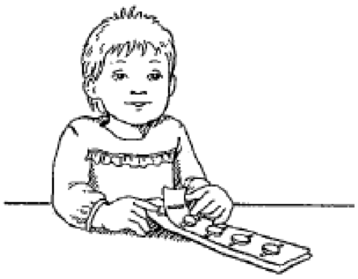
Item 24- Buttoning button<br>**Edad: 47-48 meses**<br>Posición: Sedestación<br>Estimulo: Banda de botones y cronometro<br>Procedimiento: Ponga la tira de botones en la mesa y desabroche todos los botones. Señale uno de los botones de los extremos y diga: "abrocha y desabrocha este botón tan deprisa como puedas"<br>Criterio<br>2 Abrocha y desabrocha 1 botón en 20 o menos seg<br>1 Abrocha y desabrocha 1 botón en 21 o mas seg<br>0 Agarra ambas bandas a la vez	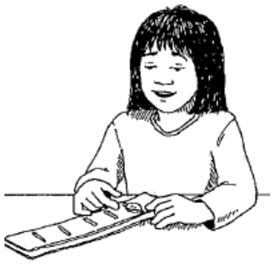
Item 25- Grasping Marker<br>**Edad: 49-50 meses**<br>Posición: Sedestación<br>Estimulo: Boligrafo y papel<br>Procedimiento: Ponga el **rotulador** y el **folio** en la mesa junto a la mano del niño. Diga: "dibuja una raya en el papel". Observe cómo agarra el rotulador.<br><br>Criterio<br>2 Agarra el rotulador con el dedo pulgar y la yema del dedo índice,	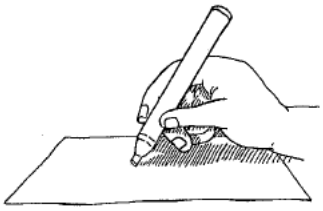

apoya el rotulador sobre la primera articulación del dedo medio<br>1 Agarra el rotulador con el dedo pulgar y la yema del dedo índice, apoyándose sobre el primer nudillo o la yema del dedo medio<br>0 Agarra el rotulador con los dedos pulgar e índice	

VISUOMOTOR COORDINATION

Material: Dos cubos, tablero, juguete atado a cuerda, taza, bolitas de comida, tupper y mas cubos, libro, botella, cuchara , tijeras, cuerda y cubos de ensartar y tablero de figuras geometricas y figuras

SUBTEST VISUOMOTOR COORDINATION PDMS2

**Item 1**<br>**Edad: 1 mes**<br>Posición: Supino<br>Estímulo: Sonajero<br>Procedimiento: Coloque al niño en decúbito supino. Quédese de pie con los pies del niño frente a la línea media de su cuerpo. Atraiga su atención sujetando un sonajero a unos 30 cm de la nariz del niño. Muevalo lentamente hacia un lado describiendo un arco de 90 grados (casi hasta tocar la superficie). Vuelva a colocarlo en la línea media y repita este procedimiento al lado contrario<br><br>Criterio<br>2- Sigue la trayectoria del sonajero 90 grados hacia cada uno de los lados<br>1-Sigue la trayectoria del sonajero menos de 90 grados hacia uno o los dos lados<br>0-Los ojos del niño se fijan en el sonajero durante 3 o menos seg.	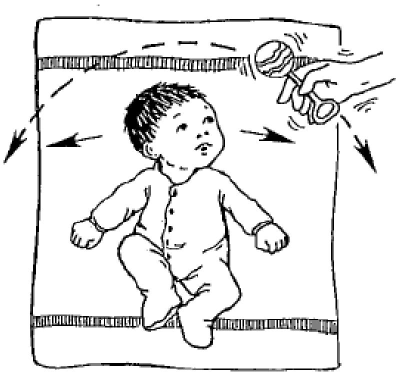
**Item 2**<br>**Edad: 1 mes**<br>Posición:Supino<br>Estímulo: Sonajero<br>Procedimiento: Coloque al niño en decúbito supino con la cabeza girada hacia un lado. Sujete un sonajero a unos 30 cm de la nariz del niño. Atraiga su atención hacia él y muévalo lentamente hacia la línea media describiendo un arco y manteniendo el sonajero a unos 30 cm de la cara del niño. Objserve los movimientos de los ojos y la rotación de la cabeza. Repita esta procedimiento hacia el otro lado.	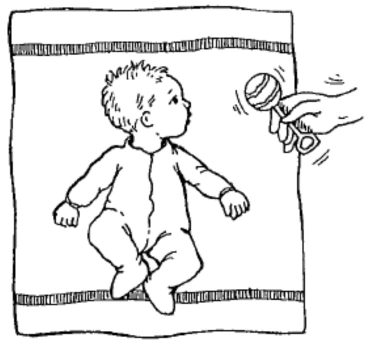

Criterio<br>2-Sigue la trayectoria del sonajero hacia la línea media con ambos lados<br>1-Sigue la trayectoria<br>0-La cabeza del niño permanece girada en un lado	
**<u>Item 3- Placing Hand</u>**<br>**Edad: 1 mes**<br>Posición: Sentado<br>Estímulo: Mesa<br>Procedimiento: Siente al niño sobre su regazo de tal forma que no este mirando hacia usted. Sientese lo suficientemente cerca de una mesa como para que el niño la alcance con comodidad. Con un movimiento hacia arriba roce con cuidado el dorso de la mano del niño con el borde de la mesa. Observe el acto reflejo de la mano que ha sido estimulada<br><br>Criterio<br>2-Coloca la mano abierta en la mesa<br>1-Coloca el puño en la mesa<br>0-No consigue colocar la mano en la mesa	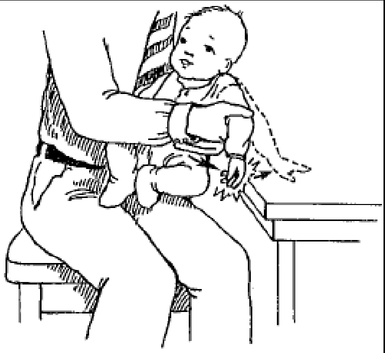
**<u>Item 4- Perceiving Rattle</u>**<br>**Edad: 2 meses**<br>Posición: Supino<br>Estímulo: Sonajero<br>Procedimiento: Coloque al niño en decúbito supino. Sujete un sonajero a unos 30 cm de la nariz del niño. Baje el sonajero lentamente hasta que esté a unos 2 cm de la nariz<br><br>Criterio<br>2-Gira la cabeza mas de 10 grados<br>1-Gira la cabeza menos de 10 grados<br>0-La cabeza del niño permanece quieta	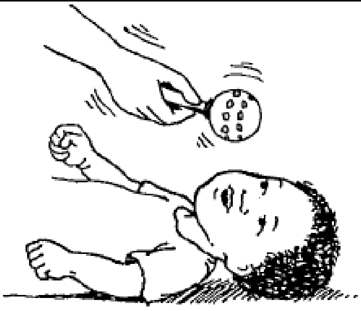
**<u>Item 5- Regarding Hands</u>**<br>**Edad: 2 meses**<br>Posición: Supino<br>Estímulo: Manos del niño<br>Procedimiento: Agarre las manos del niño y muévaselas por delante de los ojos. Observe su respuesta visual. (si el niño tiene los brazos muy cortos, gírele la cabeza hacia un lado y muévale una mano)<br><br>Criterio<br>2-Se mira las manos durante 3 seg<br>1-Se mira las manos de 1 a 2 seg<br>0-Los ojos permanecen fijos o los aparta	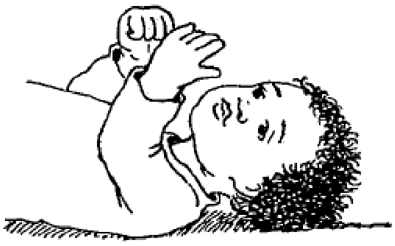
**<u>Item 6</u>**<br>**Edad: 6 meses**<br>Posición: Sedestación<br>Estímulo: Pelota de tenis<br>Procedimiento<br>Siéntese de lado junto a la mesa y coloque al niño en su regazo de frente a ésta y sujetándolo por el tronco. Atraiga su atención hacia una pelota de tenis y	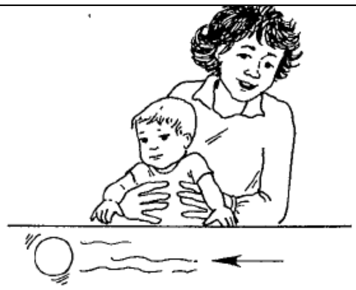

hágala rodar sobre la mesa desde el extremo izquierdo mas alejado del niño hasta el derecho. Diga: “mira la pelota”

Criterio
2-Sigue la trayectoria de la pelota más allá de la línea media
1-Sigue la trayectoria de la pelota hasta la línea media
0-La cabeza del niño permanece quieta

Item 7

Edad: 2 meses

Posición: Sentado
Estímulo: Pelota de tenis
Procedimiento: Siéntese de lado junto a la mesa y coloque al niño en su regazo de frente a ésta y sujetándolo por el tronco. Atraiga su atención hacia una pelota de tenis y hágala rodar sobre la mesa desde el extremo derecho más alejado del niño hasta el izquierdo. Diga “mira la pelota”

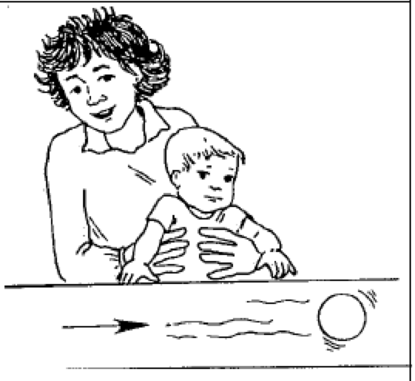

Criterio
2-Sigue la trayectoria de la pelota más allá de la línea media
1-Sigue la trayectoria de la pelota hasta la línea media
0-La cabeza del niño permanece quieta

Item 8- Tracking Rattle

Edad: 2 meses

Posición: Supino
Estimulo: Sonajero
Procedimiento: Coloque al niño en supino con la cabeza girada hacia un lado y atraiga su atención mediante un sonajero que mantendrá a unos 30 cm de la nariz del niño. Muévalo lentamente pasando la línea media, describiendo un arco de 110 grados y manteniendo el sonajero a unos 30 cm de su cara. Observe los movimientos de los ojos y la rotación de la cabeza. Vuelva a situar el sonajero en el lateral mientras continúa observando el movimiento de los ojos y de la cabeza. Repita este procedimiento hacia el lado contrario.

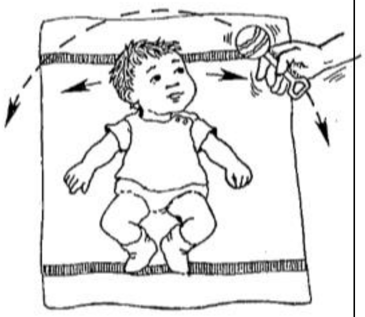

Criterio:
2-Sigue la trayectoria del sonajero a través de la línea media con ambos lados.
1-Sigue la trayectoria del sonajero a través de la línea media solamente con un lado
0-Sigue la trayectoria del sonajero hasta la línea media o menos

Item 9- Extending Arms

Edad:3 meses

Posición: Supino
Estimulo: Sonajero
Procedimiento: Coloque al niño en supino. Mueva el sonajero para atraer su atención y después sujete dicho sonajero a unos 30 cm del pecho del niño y fuera de su alcance. Diga: “Coge el sonajero”

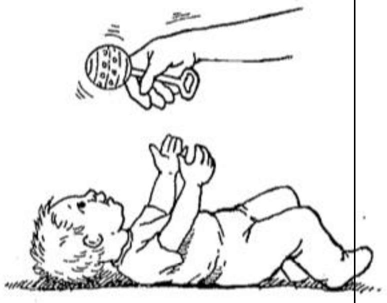

Criterio:
2-Extiende los brazos rectos hacia el sonajero

1-Extiende los brazos doblados (en un ángulo de 90 o menos grados) hacia el sonajero, o bien reacciona con el sonajero pero extiende los brazos en otra dirección
0-Los brazos del niño permanecen en la misma posición o continúan con el mismo movimiento que estaban realizando

Item 10- Approaching Midline

Edad:4 meses

Posición: Tumbado
Estimulo: Juguete atado a una cuerda
Procedimiento: Coloque al niño en decúbito supino. Haga oscilar un juguete atado a una cuerda y situado a unos 30 cm del pecho del niño. Diga "Coge el juguete"

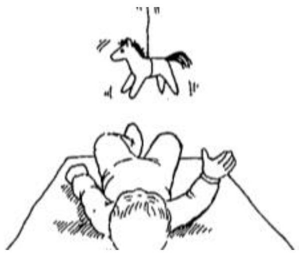

Criterio:
2-Mueve al menos 1 mano a unos 10 cm de la línea media mientras intenta alcanzar el juguete
1-Mueve al menos 1 mano en cualquier dirección, salvo hacia la línea media
0-No consigue mover ni siquiera una mano

Item 11- Fingering Hands

Edad:4 meses

Posición: Supino
Procedimiento: Coloque al niño en decúbito supino. Con las manos en la parte inferior de los brazos del niño (entre la muñeca y el codo), colóquele las manos en la línea media de manera que los dedos de ambas se toquen. Aparte las manos y observe las del niño.

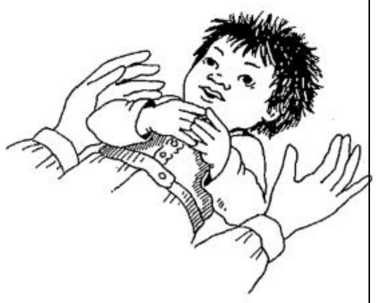

Criterio:
2-Mantiene los dedos entrelazados durante 5 seg
1-Mantiene los dedos entrelazados de 3 a 4 seg
0-Mantiene los dedos entrelazados de 0 a 2 seg

Item 12-Bringing Hands Together

Edad: 6 meses

Posición: Sentado
Estimulo: Cubo
Procedimiento: Sientese a la mesa y coloque al niño mirando hacia la misma y sentado sobre su regazo. Coloque un cubo en una de las manos del niño. Diga "juega con el cubo"

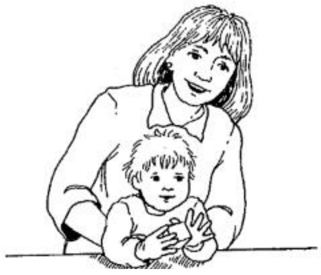

Criterio:
2-Junta las manos y agarra el cubo durante 15 seg
1-Junta las manos y agarra el cubo de 1 a 14 seg
0-No consigue juntar las manos

Item 13- Extending Arm

Edad: 6 meses

Posición: Supino
Estimulo: Sonajero

Procedimiento: Coloque al niño en supino. Mueva un sonajero y después sujételo a unos 30 cm de la nariz del niño. Diga “Coge el sonajero”

Criterio:
2-Extiende un brazo manteniendo el codo en un angulo de mas de 90 grados mientras el otro brazo permanece quieto
1-Extiende 1 brazo manteniendo el codo en un angulo de menos de 90 grados mientras el otro brazo permanece quieto
0-Extiende ambos brazos hacia el sonajero

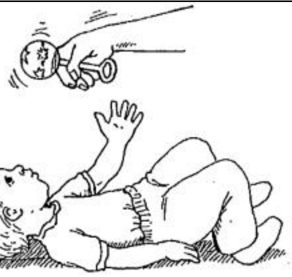

Item 14-Retaining cubes

Edad: 6 meses

Posición: Sentado
Estimulo:2 cubos
Procedimiento: Sientese a la mesa y coloque al niño mirando hacia la misma y sentado sobre su regazo. Ponga un cubo en la mesa y diga “coge el cubo”. Después de que el niño haya cogido el cubo, ponga otro cubo en la mesa. Diga “coge este también”
Criterio:
2-Coge el segundo cubo y retiene ambos cubos durante 5 seg
1-Coge el segundo cubo y retiene ambos durante menos de 5 seg
0-Solo coge un cubo

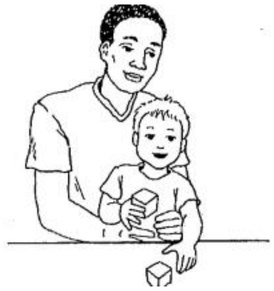

Item 15- Transfering Cube

Edad:7 meses

Posición: Sentado
Estimulo:2 cubos
Procedimiento: Sientese a la mesa y coloque al niño mirando hacia la misma y sentado sobre su regazo. Ponga un cubo en la mano del niño y otro en la mesa al alcance de la mano que ya tiene el primer cubo y tan lejos como sea posible de la mano que está libre. Diga ”Coge este también”

Criterio
2-Cambia el cubo a la otra mano y coge el segundo con la primera mano
1-Cambia el cubo a la otra mano y extiende una de las dos hacia el segundo cubo
0-Se acerca para coger el segundo cubo sin haber cambiado el primero de mano

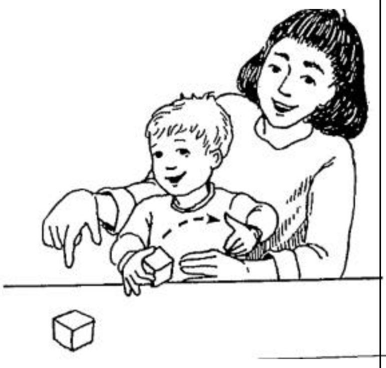

Item 16- Touching Pellet

Edad:7 meses

Posición:Sentado
Estimulo: bolitas de comida
Procedimiento: Sientese a la mesa y coloque al niño mirando hacia la misma y sentado sobre su regazo. Ponga una bolita de cereales en la mesa al alcance del niño. Diga “Coge la comida”

Criterio:
2-Toca la bolita con uno o mas dedos
1-Toca la bolita con la palma de la mano o toca la mesa cerca de la bolita
0-Extiende la mano hacia la bolita

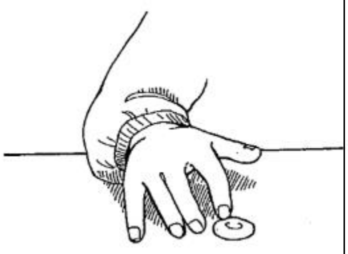

<table>
<tr><td>

<u>Item 17- Banging Cup</u>

Edad: 7 meses

Posición: Sentado
Estimulo: Taza
Procedimiento: Sientese a la mesa y coloque al niño mirando hacia la misma y sentado sobre su regazo. Atraiga la atención del niño hacia la taza que usted sujeta. Dé 3 golpes con la taza en la mesa y después déjela sobre esta. Diga “Golpea la mesa con la taza”

Criterio:
2-Da 3 golpes con la taza
1-Da 1 o 2 golpes con la taza
0-Coge la taza pero no da golpes con ella

</td><td>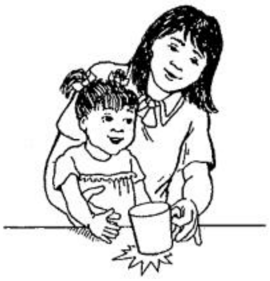</td></tr>
<tr><td>

<u>Item 18- Poking Finger</u>

Edad: 8 meses

Posición: Sentado
Estimulo: tablero
Procedimiento: Sientese a la mesa y coloque al niño mirando para la misma. Ponga un tablero de encaje de piezas vacío en la mesa delante del niño. Muestre al niño como mete el dedo índice en un agujero. Diga “Hazlo tú”

Criterio:
2-Mete el dedo índice en un agujero
1-Coloca el dedo a unos 6 mm de un agujero
0-Toca la mesa o el tablero

</td><td>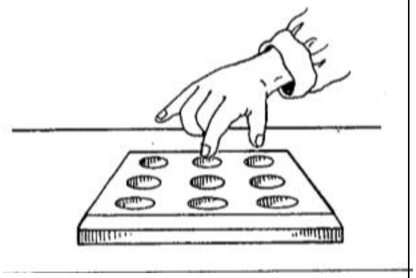</td></tr>
<tr><td>

<u>Item 19- Removing Pegs</u>

Edad:8 meses

Posición: Sentado
Estimulo: Tablero con 3 piezas ligeramente introducidas o flojas
Procedimiento: Sientese a la mesa y coloque al niño mirando hacia la misma y sentado sobre su regazo. Ponga en la mesa delante del niño un tablero con 3 piezas ligeramente introducidas. Diga “Coge las piezas”

Criterio:
2-Extrae 1 o mas piezas
1-Intenta extraer 1 pieza
0-Toca las piezas

</td><td>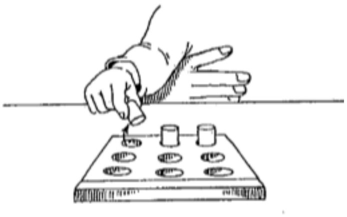</td></tr>
</table>

<table>
<tr><td>
<u>Item 20- Combining cubes</u>
Edad: 9 meses
Posición: Sedestación
Estímulo: 2 cubos

Procedimiento: Siéntese en la mesa y coloque al niño mirando hacia la misma, sentado sobre su regazo. Ponga un cubo en la mano izquierda del niño y otro cerca de la mano derecha del niño. Diga: ”coge este también y golpeándolos uno contra otro”. Hágalo usted antes si es necesario.

Criterio
2-Agarra el segundo cubo y junta ambos en la línea media
1-Coge el segundo cubo pero no consigue juntarlos en la línea media
0-No consigue agarrar el segundo cubo
</td><td>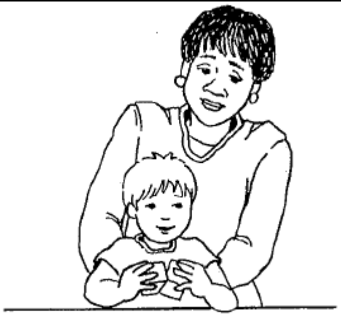</td></tr>
<tr><td>
<u>Item 21- Clapping hands</u>
Edad: 9 meses
Posición: Sentado
Procedimiento: Siente al niño mirando hacia usted y aplauda mientras dice:”haz palmitas”o “aplaude”.

Criterio
2-Da 3 palmadas
1-Da 1 o 2 palmadas
0-Junta las manos
</td><td>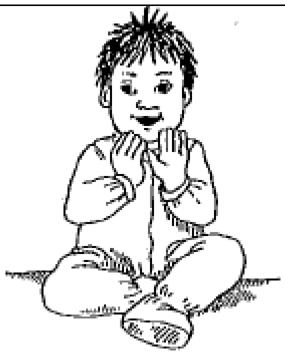</td></tr>
<tr><td>
<u>Item 22</u>- Retaining cubes
Edad: 10 meses
Posición: Sentado
Estímulo: 3 cubos

Procedimiento: Sientese a la mesa y coloque al niño mirando hacia la misma y sentado sobre su regazo. Ponga un cubo en cada mano del niño. Cuando el niño haya retenido los cubos durante 3 segundos ponga un tercer cubo en la mesa. Diga: ”Coge este también. Coge todos los cubos”

Criterio
2-Estira la mano hacia el tercer cubo mientras sujeta los otros dos
1-Deja caer uno de los cubos mientras estira la mano hacia el tercero
0-Mira el cubo
</td><td>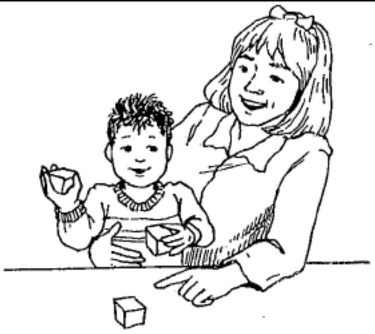</td></tr>
<tr><td>
<u>Item 23: Manipulating String</u>
Edad: 10 meses
Posición: Sedestación
Estímulo: Juguete atado a una cuerda

Procedimiento: Sientese a la mesa y coloque al niño mirando hacia la misma y sentado sobre su regazo. Ponga la cuerda en la mesa de manera que el juguete quede colgando por debajo del nivel de la misma y fuera de la vista. Diga: ”Coge la cuerda”.
</td><td>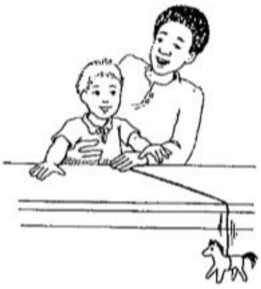</td></tr>
</table>

Criterio
2-Agarra la cuerda y tira de ella
1-Da palmaditas sobre la cuerda/acaricia la cuerda
0-Toca la cuerda

Item 24- Removing pegs

Edad: 10 meses

Posición; Sedestación
Estimulo; Tablero con tres piezas

Procedimiento: Siéntese a la mesa y coloque al niño mirando hacia la misma y sentado sobre su regazo. Ponga en la mesa delante del niño un tablero con 3 piezas ligeramente introducidas. Diga: “saca las piezas”. Si es es necesario, sujete el tablero para que no se mueva.

Criterio
2-Saca 3 piezas
1-Saca 2 piezas
0-No saca ninguna pieza o saca 1

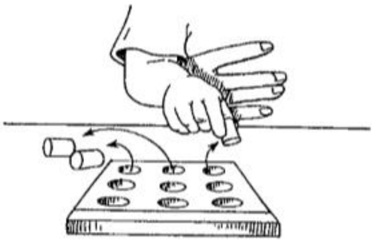

Item 25- Releasing Cube

Edad: 10 meses

Posición: Sedestación
Estimulo: Cubo

Procedimiento: Siéntese a la mesa y coloque al niño mirando hacia la misma, sentado sobre su regazo. Ponga un cubo en la mano del niño. Diga: “suelta el cubo en mi mano”. Mantenga la mano a un lado de la del niño y 15 cm por debajo de ella.

Criterio
2-Tira el cubo en la mano del examinador
1-Tira el cubo encima de la mesa
0-No tira el cubo

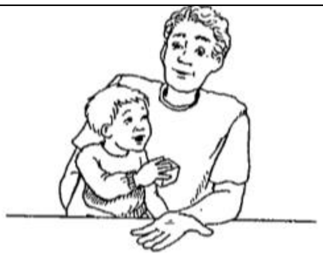

Item 26- Removing socks

Edad: 11 meses

Posición: Sedestación
Estimulo: Calcetines

Procedimiento: Siente al niño en el suelo y quítele los zapatos, diga: ”quítate los calcetines”

Criterio
2-Se quita los dos calcetines
1-Se quita un calcetín
0-Intenta quitarse un calcetín o toca los dos

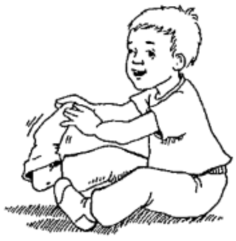

Item 27- Placing pellet

Edad: 11 meses

Posición: Sedestación
Estimulo: bolita de comida y taza

Procedimiento: Siéntese a la mesa y coloque al niño mirando hacia la misma y sentado sobre su regazo. Deje una bolita de ocmida y una taza en la mesa. Señale la bolita y diga: “métela dentro de la taza”.
Criterio
2-Coge la bolita con los dedos pulgar e índice y la deja caer dentro de la taza
1-Coge la bolita con los dedos pulgar e índice y estira la mano hacia la taza
0-Coge la bolita

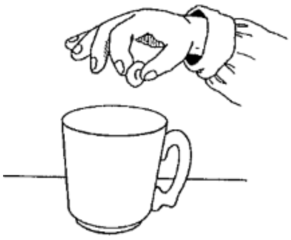

Item 28- Placing cubes

Edad: 11 meses

Posición: Sedestación
Estímulo: 7 cubos y una taza

Procedimiento: Siéntese a la mesa y coloque al niño mirando hacia la misma y sentado sobre su regazo. Ponga 7 cubos y una taza en la mesa delante del niño. Diga:” Mete los cubos dentro de la taza”.

Criterio
2-Introduce de 3 a 7 cubos dentro de la taza
1-Introduce de 1 a 2 cubos dentro de la taza
0-No consigue introducir ningún cubo dentro de la taza

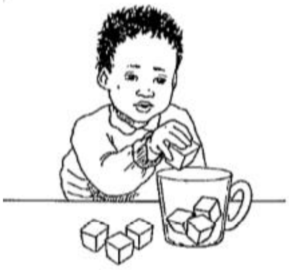

Item 29- Turning pages

Edad: 12 meses

Posición: Sentado
Estimulo: Libro con pastas y paginas gruesas

Procedimiento: Sientese a la mesa con el niño sentado sobre su regazo en una posición segura frente a la mesa. Ponga el libro con pastas y paginas gruesas en la mesa y diga “abre el libro”.

Criterio
2-Abre el libro
1-Intenta abrir el libro
0-Da palmaditas en el libro

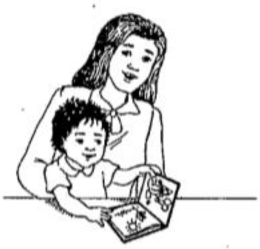

Item 30- Stirring spoon

Edad: 12 meses

Posición: Sentado
Estimulo: Cuchara y taza

Procedimiento: Siéntese a la mesa con el niño sentado sobre su regazo en una posición segura frente a la mesa. Muestre al niño como remover con la cuchara en la taza. Ponga la cuchara junto a la taza y diga: “remueve con la cuchara”

Criterio

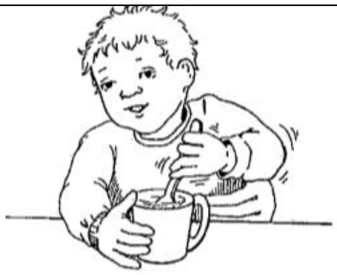

2-Da vueltas con la cuchara en la taza<br>1-Introduce la cuchara en la taza o mueve la cuchara hacia arriba y hacia abajo dentro de la taza<br>0-Agarra la cuchara	
**<u>Item 31- Removing pellets</u>**<br>**Edad: 12 meses**<br>Posición: Sentado<br>Estimulo: botella sin tapón con una bolita de comida dentro<br>Procedimiento: Siéntese a la mesa con el niño sentado sobre su regazo en una posición segura frente a la mesa. Entregue al niño una botella con una bolita dentro. Diga: “saca la bolita”<br>Criterio<br>2-Da la vuelta a la botella y saca la bolita<br>1-Intenta sacar la bolita<br>0-Agarra la botella	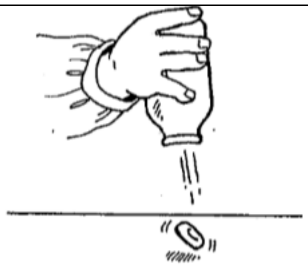
**<u>Item 32-Placing cubes</u>**<br>**Edad: 13 meses**<br>Posición: Sentado<br>Estímulo: 7 cubos y una taza<br><br>Procedimiento: Siéntese a la mesa con el niño sentado sobre su regazo en una posición segura frente a la mesa. Ponga 7 cubos y una taza en la mesa enfrente del niño. Diga “mete los cubos dentro de la taza”<br><br>Criterio<br>2-Introduce 7 cubos dentro de la taza<br>1-Intrduce de 4 a 6 cubos dentro de la taza<br>0-Introduce de 0 a 3 cubos dentro de la taza	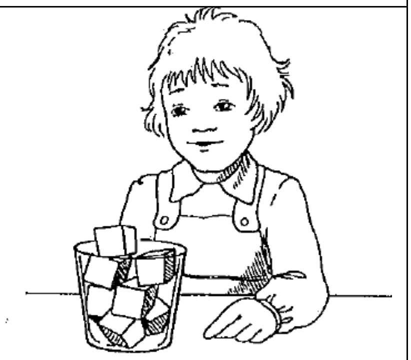
**<u>Item 33-Placing pegs</u>**<br>**Edad: 13 meses**<br>Posición: Sentado<br>Estimulo: Tablero y 3 piezas<br><br>Procedimiento: Siéntese a la mesa con el niño sentado sobre su regazo en una posición segura frente a la mesa. Ponga el tablero en la mesa delante del niño. Coloque 3 piezas encima de la mesa entre el niño y el tablero. Diga: “Coloca las piezas en el tablero”<br><br>Criterio<br>2-Coloca 3 piezas en el tablero<br>1-Coloca 1 o 2 piezas en el tablero<br>0-Coge las piezas	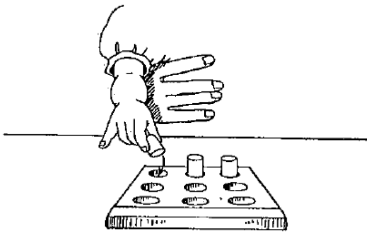

Item 34-Taping spoon

Edad: 13 meses

Posición: Sentado
Estimulo: Cuchara y taza

Procedimiento: Siéntese a la mesa con el niño sentado sobre su regazo en una posición segura frente a la mesa. Muestre al niño como golpear la taza con la cuchara con un movimiento horizontal. Coloque la cuchara en la mesa. Diga “ahora lo haces tú”

Criterio
2-Golpea la taza con un movimiento horizontal
1-Golpea la taza con un movimiento vertical
0-Coge la cuchara

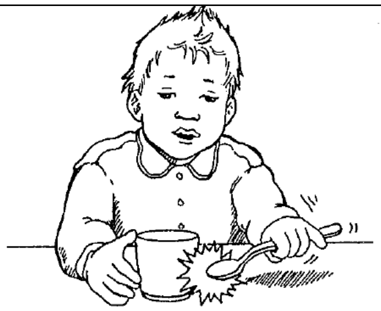

Item 35-Inserting Shapes

Edad:13 meses

Posición: Sentado
Estimulo: Tablero de encaje y figuras geométricas

Procedimiento: Siéntese a la mesa y coloque al niño mirando hacia la misma y sentado sobre su regazo. Ponga el tablero de encaje de figuras geométricas en la mesa delante de niño y las figuras entre el niño y dicho tablero; asegúrese de que dichas figuras están alineadas con los agujeros en los que tiene que introducirlas. Señale las figuras y los agujeros y diga; “Coloca las figuras en el tablero”

Criterio
2-Coloca 1 figura en el agujero correcto
1-Coloca 1 figura en el agujero correcto de forma parcial
0-Coge 1 figura y la coloca sobre el tablero

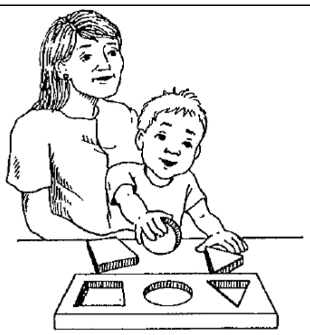

Item 36-Placing pellet

Edad: 14 meses

Posicion: Sentado
Estimulo: 4 bolitas de comida y una botella

Procedimiento: Siéntese a la mesa con el niño sentado sobre su regazo en una posición segura frente a la mesa. Ponga la botella y las 4 bolitas encima de la mesa delante del niño. Coja una bolita y métala dentro de la botella. Señale otra bolita y diga; “mete la bolita dentro de la botella”

Criterio
2-Introduce la bolita dentro de la botella
1-Intenta introducir una bolita dentro de la botella
0-Coge la bolita

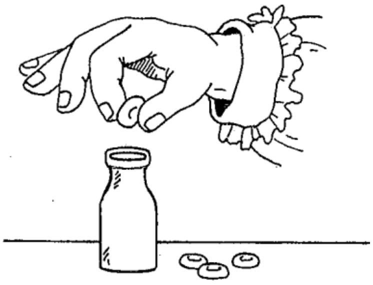

Item 37-Scribbling

Edad: 14 meses

Posición: Sentado
Estimulo: 2 rotuladores y 2 folios

Procedimiento: Siéntese a la mesa con el niño sentado sobre su regazo en una posición segura frente a la mesa. Utilice un rotulador para trazar 2 líneas verticales paralelas de unos 7 cm de longitud en un folio. Ponga otro folio y otro rotulador en la mesa delante del niño. Diga “haza lo que hice yo”

Criterio
2-Hace al menos un garabato de más de 2 cm de longitud
1-Hace un garabato de menos de 2 cm de longitud
0-Toca el papel con el rotulador

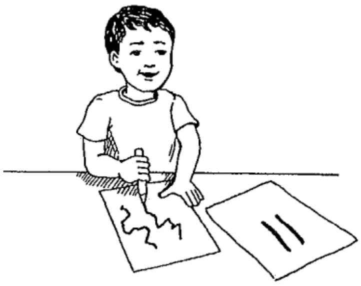

Item 38-Building tower

Edad: 15-16 meses

Posición: Sentado
Estimulo: 6 cubos

Procedimiento: Siéntese a la mesa con el niño sentado sobre su regazo en una posición segura frente a la mesa. Diga: “mira cómo construyo una torre”. Coja un cubo y colóquelo con cuidado sobre otro cubo de forma que los lados estén alineados. Coja otro cubo y colóquelo con cuidado sobre el segundo. Deje la torre en pie y ponga 3 cubos delante del niño. Diga: “ahora tú vas a construir una torre”

Criterio
2-Construye una torre de 2 o 3 cubos
1-Intenta apilar 2 cubos
0-Coge un cubo

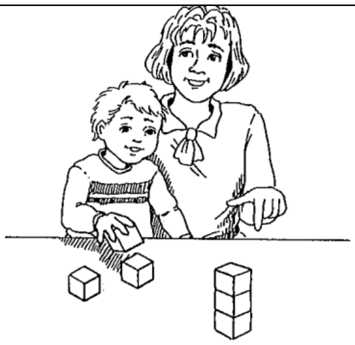

Item 39-Inserting Shapes

Edad:17-18

Posición: Sentado
Estimulo: Tablero de encaje y figuras geométricas

Procedimiento: Siéntese a la mesa y coloque al niño mirando hacia la misma y sentado sobre su regazo. Ponga el tablero de encaje de figuras geométricas en la mesa delante del niño y las figuras entre el niño y dicho tablero, pero no alineadas con los agujeros en los que tiene que introducirlas. Señale las figuras y después los agujeros y diga: “coloca las figuras en el tablero”.

Criterio
2-Coloca 2 figuras en los agujeros correctos
1-Coloca una figura en el agujero correcto y la segunda figura de manera parcial en el agujero correcto
0-Coloca 1 figura en el agujero correcto

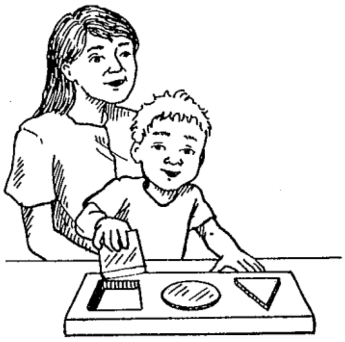

Item 40-Building Tower

Edad:19-20 meses

Posición: Sentado
Estimulo: 10 cubos

Procedimiento: Siéntese a la mesa con el niño sentado sobre su regazo en una posición segura frente a la mesa. Diga: “mira cómo construyo una torre alta”. Coja un cubo y colóquelo con cuidado sobre otro cubo de forma que los lados estén alineados. Coja otro cubo y colóquelo con cuidado sobre el segundo. Continúe apilando los cubos hasta tener una torre de 5. Deje la torre en pie y ponga 5 cubos delante del niño. Diga: “ahora tú vas a construir una torre”

Criterio
2-Apila 4 o 5 cubos
1-Apila 3 cubos
0-Apila 2 cubos

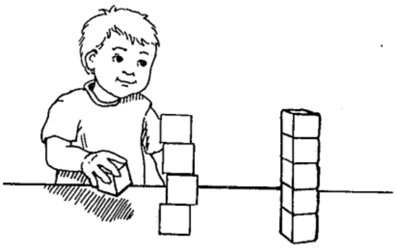

Item 41-Turning pages

Edad:19-20 meses

Posición: Sentado
Estimulo: Libro con pastas y páginas gruesas

Procedimiento: Siéntese a la mesa y coloque al niño mirando hacia la misma y sentado sobre su regazo. Ponga un libro con las pastas y las páginas gruesas en la mesa delante del niño. Diga: “mira el libro”.

Criterio
2-Pasa 3 páginas de una en una
1-Pasa 2 páginas de una en una o pasa 2 o más paginas juntas
0-Abre el libro

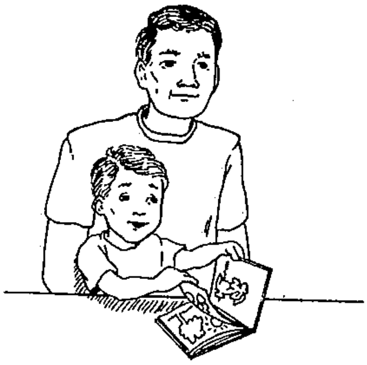

Item 42-Inserting Shapes

Edad: 19-20 meses

Posición: Sentado
Estimulo: Tablero de encaje de figuras y figuras geométricas

Procedimiento: Siéntese a la mesa y coloque al niño mirando hacia la misma y sentado sobre su regazo. Ponga el tablero de encaje de figuras geométricas en la mesa delante del niño y 3 figuras entre el niño y dicho tablero, pero no alineadas con los agujeros en los que tiene que introducirlas. Señale las figuras y después los agujeros. Diga: “coloca las figuras en el tablero”

Criterio
2-Coloca 3 figuras en los agujeros correctos
1-Coloca 2 figuras en los agujeros correctos y una tercera de forma parcial
0-Coloca 2 figuras en los agujeros correctos

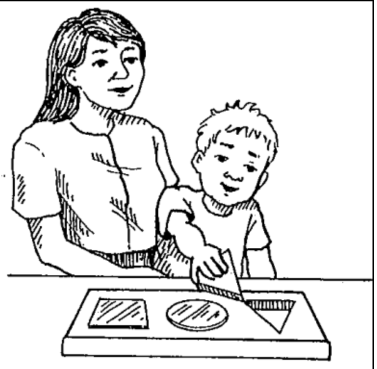

Item 43-Building tower

Edad: 21-22 meses

Posición: Sentada
Estimulo: 6 cubos

Procedimiento: Siéntese a la mesa con el niño sentado sobre su regazo en una posición segura frente a la mesa. Ponga 6 cubos en la mesa. Diga: “mira como construyo una torre alta”. Coja un cubo y colóquelo con cuidado sobre otro cubo de forma que los lados estén alineados. Coja otro cubo y colóquelo con cuidado sobre el segundo. Continúe apilando los cubos hasta tener una torre de 6. Deje la torre en pie durante unos segundos, derríbela y dele 6 cubos al niño. Diga:” ahora tú vas a construir una torre alta”

Criterio
2-Apila 6 cubos
1-Apila 5 cubos
0-Apila 4 cubos

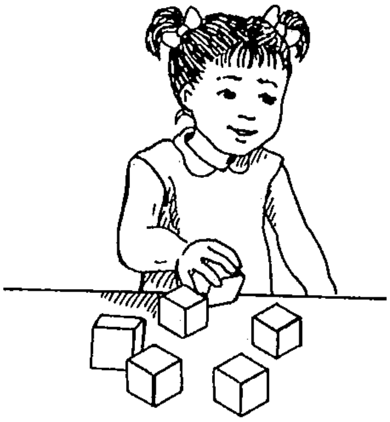

Item 44-Imitating Vertical Strokes

Edad:23-24 meses

Posición: Sentado
Estimulo: 2 rotuladores y 2 folios

Procedimiento: Siéntese a la mesa y coloque al niño mirando hacia la misma y sentado sobre su regazo. Trace 2 líneas verticales de unos 7 cm de longitud en un folio. Ponga otro folio y un rotulador en la mesa delante del niño. Diga: “dibuja una línea de arriba abajo como yo hice”

Criterio
2-Traza una línea de unos 5 cm de longitud y sin desplazarse más de 20 grados de la vertical
1-Traza una línea de unos 5 cm de longitud sin desplazarse más de entre 21 y 45 grados de la vertical
0-La línea trazada tiene una longitud de menos de 5 cm o está a más de 45 grados de la vertical

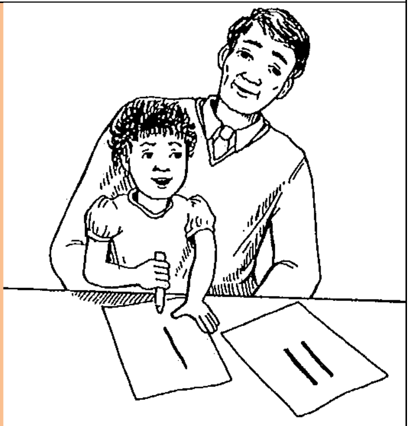

Item 45-Removing top

Edad:25-26 meses

Posición: Sentado
Estimulo: Una botella con tapón de rosca y una bolita de comida

Procedimiento: Ponga una bolita de comida dentro de la botella y enrosque el tapón. Entregue la botella al niño y diga:” coge la comida”

Criterio
2-Desenrosca el tapón
1-Intenta desenroscar el tapón
0-Agita la botella

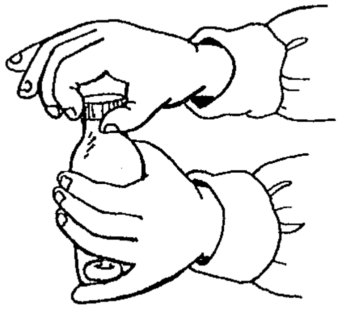

Item 46-Building Tower

Edad:25-26 meses

Posición: Sentado
Estimulo: 10 cubos

Procedimiento: Ponga 10 cubos encima de la mesa. Diga “mira como construyo una torre alta”. Coja un cubo y colóquelo con cuidado sobre el segundo. Continúe apilando los cubos hasta tener una torre de 10. Deje la torre en pie durante unos segundos, derríbela y ponga los 10 cubos delante del niño. Diga: “ahora tú vas a construir una torre alta”

Criterio
2-Apila 8 cubos
1-Apila 7 cubos
0-Apila 6 cubos

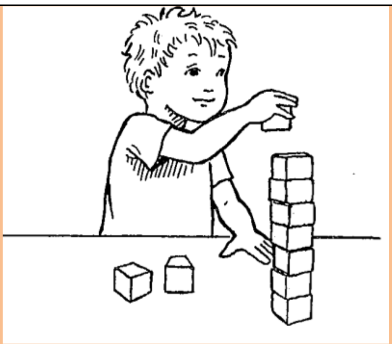

Item 47-Snipping with Scissors

Edad: 25-26 meses

Posición: Sentado
Estimulo: Tijeras romas y papel

Procedimiento: Coloque las manos de forma que el niño vea con claridad lo que está haciendo y muéstrele como recortar el borde de un papel por tres lugares distintos con unas tijeras romas. Entregue el papel y las tijeras al niño y diga: “ahora tú vas a recortar el papel”.

Criterio
2-Corta el papel por un lugar
1-Abre las tijeras e intenta cortar
0-Toca el papel con las tijeras

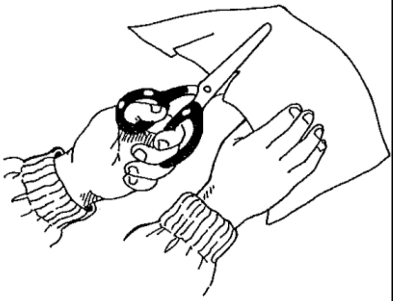

Item 48-Imitating Horizontal Strokes

Edad:27-28 meses

Posición: Sentado
Estimulo: 2 rotuladores y 2 folios

Procedimiento: Trace 2 líneas horizontales de unos 7 cm de longitud en un folio. Ponga otro folio y un rotulador en la mesa delante del niño. Diga: “dibuja una línea como yo hice”

Criterio
2-Traza una línea de unos 5 cm de longitud sin desplazarse más de 20 grados del plano horizontal
1-Traza una línea de unos 5 cm de longitud sin desplazarse más de entre 21 y 45 grados del plano horizontal
0-La línea trazada tiene una longitud de menos de 5 cm o está a más de 45 grados del plano horizontal

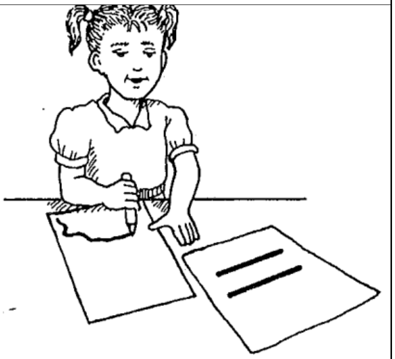

Item 49-Strinting Beads

Edad: 27-28 meses

Posición: Sentado

Estimulo: Cordón y cuentas de ensartar

Procedimiento: Coloque las manos de forma que el niño vea con claridad lo que está haciendo y muéstrele como ensartar 2 cuentas en el cordón. Coja una cuenta con el agujero hacia usted y muéstrele el agujero al niño. Coja el cordón con la otra mano y páselo con cuidado por el agujero desde adelante hacia atrás. Deslice la cuenta hacia la mitad del cordón. Coja otra cuenta y engárcela del mismo modo. Entregue el cordón con las 2 cuentas al niño y ponga 4 más en la mesa. Diga: “ensarta las cuentas en el cordón como yo hice”.

Criterio

2-Ensarta 2 cuentas

1-Ensarta 1 cuenta

0-Intenta ensartar 1 cuenta

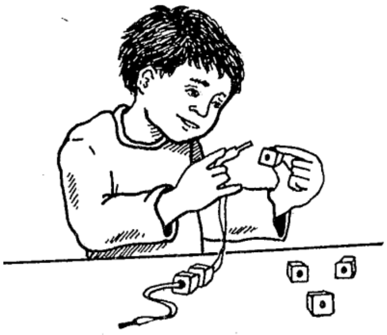

Item 50-Folding paper

Edad: 27-28 meses

Posición: Sentado

Estímulo: Papel Din A4 cortado por la mitad

Procedimiento: Coloque las manos de forma que el niño pueda ver con claridad lo que está haciendo y muéstrele como doblar medio folio a la mitad. Deje el modelo doblado a la vista del niño, entréguele la otra mitad del papel y diga: “dóblalo como el mio”

Criterio

2-Dobla el papel marcando una línea

1-Arruga el papel

0-Toca el papel

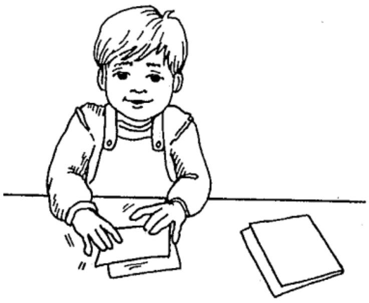

Item 51- Building train

Edad: 29-30 meses

Posición: Sentado

Estímulo: 8 cubos

Procedimiento: Ponga 4 cubos encima de la mesa y coloque las manos de forma que el niño pueda ver con claridad lo que está haciendo. Muéstrele como construir un tren con 3 cubos alineados y otro encima del primer cubo. Empuje el tren por la mesa mientras emite el ruido de una locomotora y déjelo donde el niño pueda verlo. Coloque los otros 4 cubos en la mesa delante del niño y diga: “haz un tren como el mío”

Criterio

2-Coloca 3 cubos alineados y el cuarto sobre el ultimo

1-Coloca 3 cubos alineados, pero no consigue colocar correctamente el cubo superior

0-Coloca 2 cubos alineados

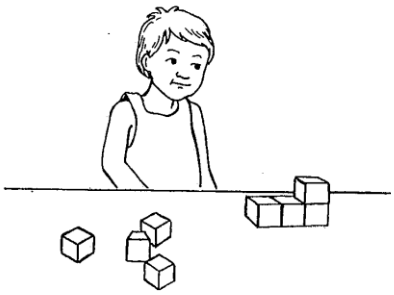

Item 52- Stringing Beads

Edad: 29-30 meses

Posición: Sentado
Estímulo: cordón y 6 cubos con agujero

Procedimiento: Coloque las manos de forma que el niño vea con claridad lo que está haciendo y muéstrele como ensartar 2 cuentas cuadradas en el cordón. Coja una cuenta con el agujero hacia usted y muéstrele el agujero al niño. Coja el cordón con la otra mano y páselo con cuidado por el agujero desde adelante hacia atrás. Deslice la cuenta hacia la mitad del cordón. Coja otra cuenta y ensartela del mismo modo. Entregue el cordón con las 2 cuentas al niño y ponga 4 más en la mesa. Diga: “ensarta todas estas cuentas en el cordón como yo hice”

Criterio
2-Ensarta 4 cuentas
1-Ensarta 3 cuentas
0-Ensarta 2 cuentas

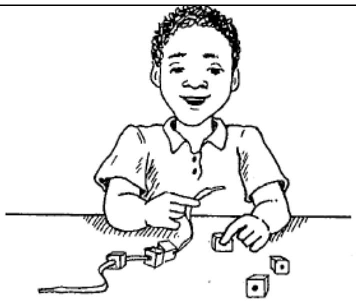

Item 53- Building tower

Edad: 29-30 meses

Posición: Sentado
Estímulo: 10 cubos

Procedimiento: Ponga 5 cubos en la mesa. Diga “mira como construyo una torre”. Coja un cubo y colóquelo con cuidado sobre otro cubo de forma que los lados estén alineados. Coja otro cubo y colóquelo con cuidado sobre el segundo. Continúe apilando los cubos hasta que los 5 formen una torre. Deje la torre en pie durante unos segundos, derríbela, ponga los 10 cubos delante del niño diga: “construye una torre alta utilizando tantos cubos como puedas”.

Criterio
2-Apila 10 cubos
1-Apila 9 cubos
0-Apila menos de 9 cubos

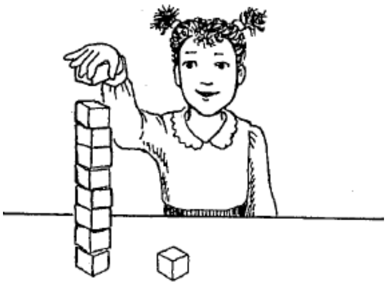

Item 54- Building bridge

Edad: 31-32 meses

Posición: Sentado
Estímulo: 6 cubos

Procedimiento: Ponga 3 cubos en la mesa y coloque las manos de forma que el niño pueda ver con claridad lo que está haciendo. Muéstrele como construir un puente como el del dibujo con 3 cubos y déjelo en pie. Ponga 3 cubos en la mesa delante del niño. Diga: “construye un puente como el mío”.

Criterio
2-Construye un puente como se muestra en la imagen
1-Construye un puente con los dos cubos de la base en contacto o con el cubo superior descolocado
0-Apila los cubos

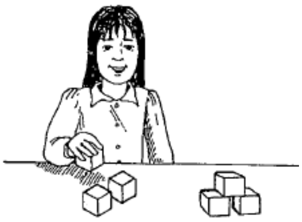

Item 55- Copying circle

Edad: 33-34 meses

Posición: Sentado

Estímulo: Rotulador, papel y un folio con un circulo ya hecho

Procedimiento: Ponga un trozo de papel y un rotulador en la mesa delante del niño. Enseñe al niño la tarjeta con el circulo y déjela en la mesa junto a la parte superior del papel. Diga: “dibuja un circulo”

Criterio

2-Dibuja un circulo con el punto final desplazado un máximo de 12mm del punto inicial

1-Dibuja al menos ¾ partes del circulo con el punto final desplazado entre 12 y 25 mm de la inicial

0-Intenta dibujar un círculo cuyo punto final está a más de 25 mm del inicial o dibuja menos de las ¾ partes del circulo

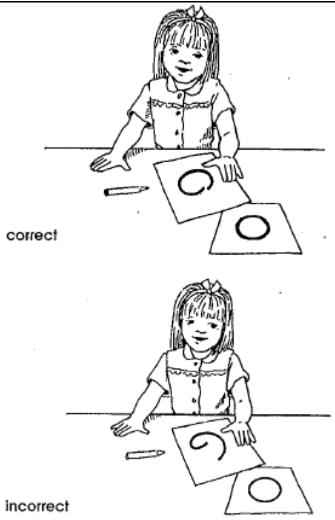

Item 56- Building wall

Edad: 35-36 meses

Posición: Sentado

Estímulo: 8 cubos

Procedimiento: Ponga 4 cubos encima de la mesa y coloque las manos de forma que el niño pueda ver con claridad lo que está haciendo. Muéstrele como construir dos torres de 2 cubos que están unidas para formar una pared y déjela en pie. Ponga otros 4 cubos en la mesa delante del niño. Diga: “construye una pared como la mía”

Criterio

2-Construye una pared o 2 torres unidas

1-Construye torres de 2 cubos, pero separadas

0-Construye una sola torre

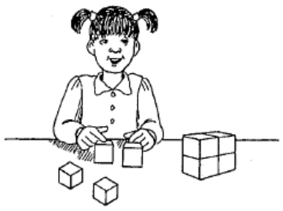

Item 57- Cutting paper

Edad: 37-38 meses

Posición: Sentado

Estímulo: 2 piezas de papel y tijeras

Procedimiento: Coloque las manos de forma que el niño vea con claridad lo que está haciendo muéstrele como cortar por la mitad uno de los 2 folios. Ponga el segundo folio y las tijeras en la mesa delante del niño. Diga: “corta el folio como hice yo”

Criterio

2-Corta el folio en 2 trozos

1-Corta ¾ o menos del folio

0-Abre y cierra tijeras varias veces

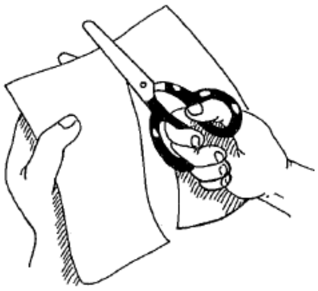

<u>Item 58- Lacing string</u>

Edad: 39-40 meses

Posición: Sentado
Estímulo: tira de agujeros y cordón

Procedimiento: Muéstrele al niño una tira con 6 agujeros. Diga: “mira como paso el cordón por los agujeros”. Coloque las manos de forma que el niño vea con claridad lo que está haciendo y pase el cordón de arriba abajo por el primer agujero, hacia arriba por el segundo y hacia abajo por el tercero. Muestre la tira al niño y quite el cordón. Entregue la banda y el cordón al niño y diga: “ahora haz lo que yo hice”

Criterio
2-Pasa el cordón por 3 agujeros
1-Pasa el cordón por 2 agujeros
0-Pasa el cordón por 1 agujero o no lo pasa por ninguno

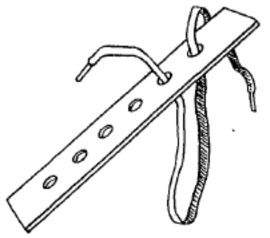

<u>Item 59- Copying cross</u>

Edad: 39-40 meses

Posición: Sentado
Estímulo: Rotulador, papel y folio con una cruz hecha

Procedimiento: Ponga un folio y un rotulador en la mesa delante del niño. Muestre al niño la tarjeta con la cruz y déjela en la mesa junto a la parte superior del folio. Diga: “dibuja líneas como estas que se crucen en el centro”

Criterio
2-Traza líneas que se cruzan y no se desvían más de 20 grados de la perpendicular
1-Traza líneas que se cruzan y se desvían más de 20 grados de la perpendicular
0-Traza líneas que no se cruzan

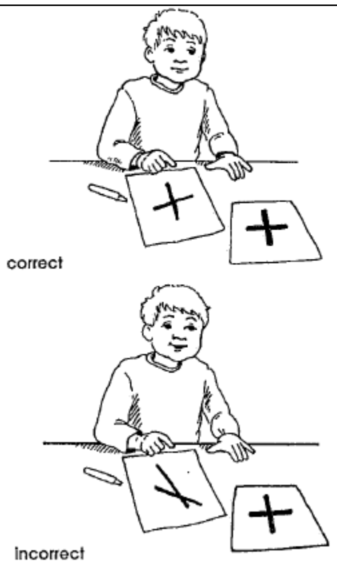

Supplement: Supplementary file 1 [file Datasheet1.pdf]
